# Structure-function analysis of the Yhc1 subunit of yeast U1 snRNP and genetic interactions of Yhc1 with Mud2, Nam8, Mud1, Tgs1, U1 snRNA, SmD3 and Prp28

**DOI:** 10.1093/nar/gku097

**Published:** 2014-01-31

**Authors:** Beate Schwer, Stewart Shuman

**Affiliations:** ^1^Microbiology and Immunology Department, Weill Cornell Medical College, New York, NY 10065, USA and ^2^Molecular Biology Program, Sloan-Kettering Institute, New York, NY 10065, USA

## Abstract

Yhc1 and U1C are homologous essential subunits of the yeast and human U1 snRNP, respectively, that are implicated in the establishment and stability of the complex of U1 bound to the pre-mRNA 5′ splice site (5′SS). Here, we conducted a mutational analysis of Yhc1, guided by the U1C NMR structure and low-resolution crystal structure of human U1 snRNP. The N-terminal 170-amino acid segment of the 231-amino acid Yhc1 polypeptide sufficed for vegetative growth. Although changing the zinc-binding residue Cys6 to alanine was lethal, alanines at zinc-binding residues Cys9, His24 and His30 were not. Benign alanine substitutions at conserved surface residues elicited mutational synergies with other splicing components. *YHC1-R21A* was synthetically lethal in the absence of Mud2 and synthetically sick in the absence of Nam8, Mud1 and Tgs1 or in the presence of variant U1 snRNAs. *YHC1* alleles *K28A*, *Y12A*, *T14A*, *K22A* and *H15A* displayed a progressively narrower range of synergies. *R21A* and *K28A* bypassed the essentiality of DEAD-box protein Prp28, suggesting that they affected U1•5′SS complex stability. Yhc1 Arg21 fortifies the U1•5′SS complex via contacts with SmD3 residues Glu37/Asp38, mutations of which synergized with *mud2*Δ and bypassed *prp28*Δ. *YHC1-(1-170)* was synthetically lethal with mutations of all components interrogated, with the exception of Nam8.

## INTRODUCTION

Yeast pre-mRNA splicing (1–3) begins with the formation of a complex comprising the U1 snRNP bound at the intron 5′ splice site (5′SS; 5′-GUAUGU) and the Msl5•Mud2 heterodimer engaged at the intron branchpoint (BP; 5′-UACUAAC). Bridging interactions between the U1 snRNP and Msl5•Mud2 stabilize the complex and prepare a scaffold for recruitment of the U2 snRNP to the branchpoint. The U1 snRNP is ultimately ejected from the pre-mRNA•U1•U2-containing spliceosome when the U5•U4•U6 tri-snRNP complex joins *en route* to forming a pre-mRNA•U2•U5•U6 spliceosome. Dissociation of U1 snRNP is thought to be triggered by the DEAD-box protein Prp28 (4*,*^5^), acting to disrupt the short U1:5′SS RNA duplex or remodel protein-RNA contacts at the 5′SS (or both).

The *Saccharomyces cerevisiae* U1 snRNP consists of a trimethylguanosine (TMG)-capped 568-nt U1 snRNA, a 7-subunit Sm protein ring (common to the U2, U4 and U5 snRNPs), and 10 protein subunits unique to the yeast U1 snRNP: Prp39, Prp40, Snu71, Snu56, Snp1, Mud1, Luc7, Prp42, Nam8 and Yhc1 (6–9). The composition of the U1 snRNP is more complex in budding yeast than in humans (10–12), with respect to the size of the U1 RNA (568 versus 164 nt) and the number of U1-specific protein subunits (10 versus 3). The three human U1-specific snRNP subunits—U1-70K, U1-A and U1-C—are homologs of yeast Snp1, Mud1 and Yhc1, respectively.

The conserved 5′ leader sequence of yeast and human U1 RNA—m^2,2,7^GpppAUACUUACC—contains a hexanucleotide motif (underlined) that is complementary to the consensus yeast 5′SS. The ACUUAC sequence pairs with the pre-mRNA to nucleate an early assembly intermediate. Although the ACUUAC motif in U1 RNA is essential for yeast viability, more than half of the U1 primary structure, including the 5′ TMG cap, is dispensable (13–17). In the same vein, the U1 snRNP subunits Mud1 and Nam8 are inessential, as is the Mud2 subunit of the Msl5•Mud2 branchpoint-binding protein (7*,*^17–21^). Substantial chunks of the essential U1 snRNP subunits Snp1 and Prp40 and the essential branchpoint-binding subunit Msl5 can also be deleted without compromising yeast viability (22–26).

This is not to say that the many dispensable elements of the yeast U1 snRNP are functionally irrelevant. Rather, traditional and genome-wide genetic analyses have highlighted a network of genetically buffered functions during early spliceosome assembly, embracing the U1 snRNP, the Msl5•Mud2 branchpoint-binding protein, the TMG cap and the Cbc2•Sto1 nuclear m^7^G cap-binding complex (7*,*^17–34^). This network was defined by the numerous instances in which null alleles of inessential players, or benign mutations in essential factors, elicited synthetic lethal phenotypes when combined with other benign mutations in the splicing machinery. Such genetic interactions among actors in a common pathway meet an operational definition of redundancy, which does not necessitate that the synthetic interactor proteins or RNA elements perform the same task, but rather suggests that spliceosome assembly can be accomplished or stabilized via different sub-pathways.

Although mutational synergies have played a decisive role in identifying many of the components of the yeast spliceosome (7*,*^18^*,*^19^*,*^27^*,*^29^), the interpretation of synthetic phenotypes can be elusive, especially when the atomic structures or biochemical activities of the genetically interacting factors are unknown. However, when structures are available, they can be exploited to program mutations with specific functional defects and then systematically test an allelic series for synthetic genetic interactions with other spliceosome components or splicing factors. This has been applied to the m^7^G-cap binding pocket of yeast Cbc2 (34), guided by the crystal structure of the homologous human CBC•m^7^G-cap complex (35*,*^36^), and to the branchpoint RNA-binding site of yeast Msl5 (25*,*^26^), directed by the NMR structure of the human homolog SF1 bound to an RNA containing the yeast branchpoint consensus sequence (37).

**Figure 1.**
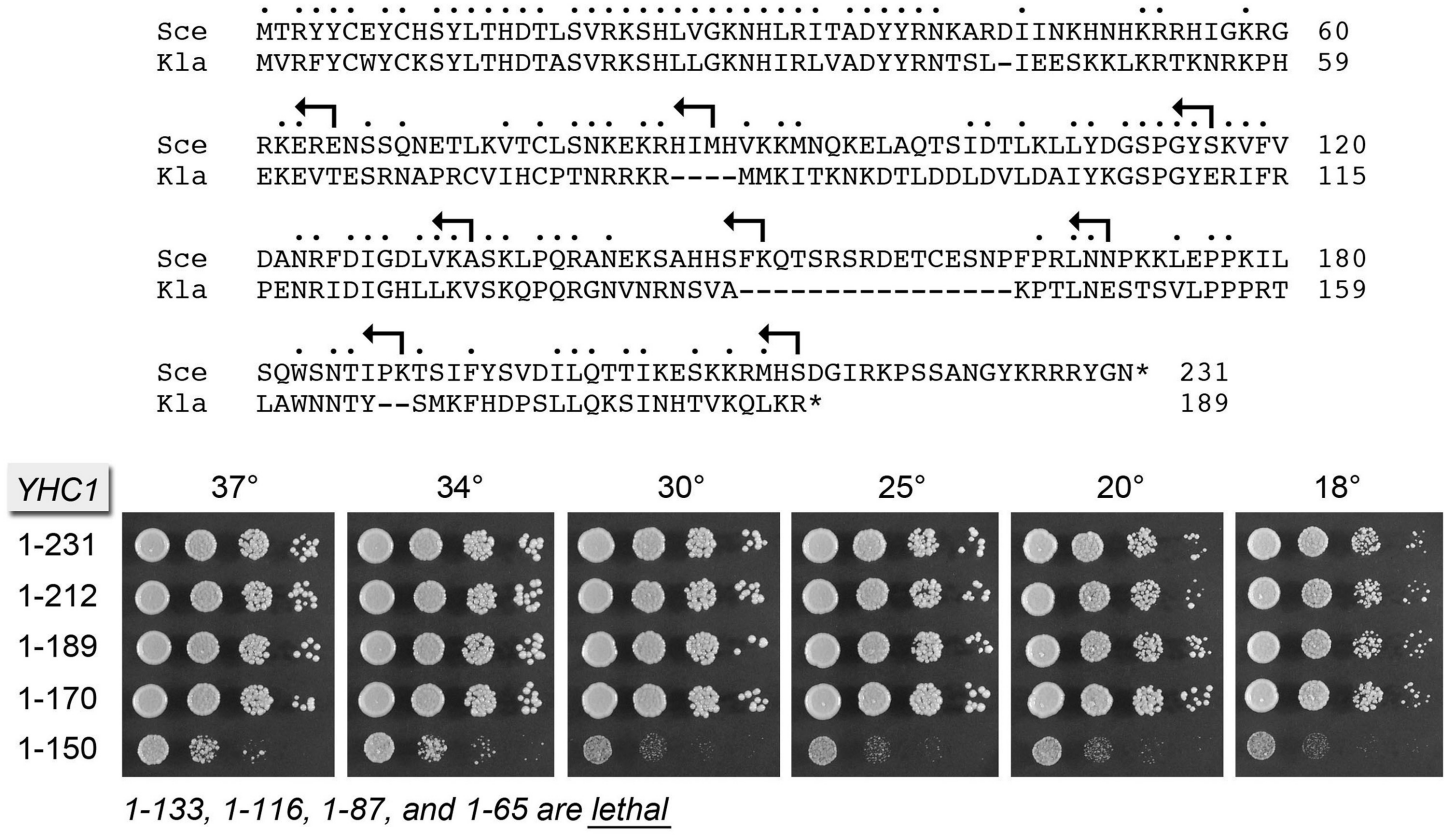
Effects of C-terminal truncations on Yhc1 activity *in vivo*. (Top panel) The amino acid sequence of *Saccharomyces cerevisiae* (Sce) Yhc1 is aligned to that of the homologous protein from *Kluyveromyces lactis* (Kla). Positions of amino acid site chain identity/similarity are denoted by • above the sequence. Reverse arrowheads indicate the boundaries of the C terminal truncations of Yhc1. (Bottom panel) The wild-type and truncated *YHC1* alleles were tested for activity by plasmid shuffle as described under Methods. The growth phenotypes of viable FOA-resistant *yhc1*Δ p[*CEN HIS3 YHC1*] strains bearing the indicated *YHC1* alleles were assessed as follows. Liquid cultures were grown to mid-log phase at 30°C and adjusted to the same *A*_600_. Aliquots (3 µl) of serial 10-fold dilutions of cells were spotted to YPD agar. The plates were incubated at the indicated temperatures and photographed after 2 d (30, 34 and 37°C), 3 d (25°C) or 4 d (18 and 20°C). The truncated mutants listed at *bottom* failed to complement *yhc1*Δ in a plasmid shuffle assay and were deemed lethal.

**Figure 2.**
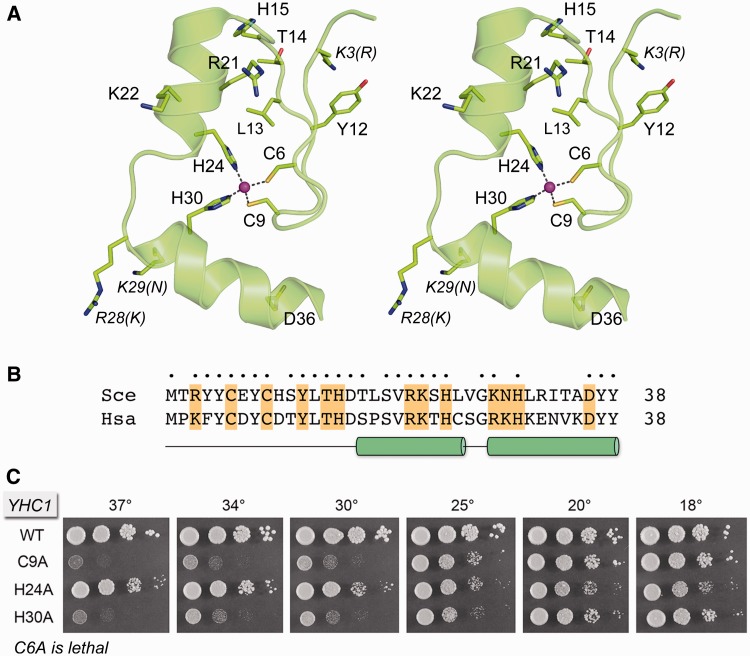
Structure-guided mutagenesis of the Yhc1 N-terminal domain. (**A**) Stereo view of the NMR structure of the N-terminal domain of human U1C (pdb 2VRD), highlighting the tetrahedral coordination complex of a Zn^2+^ ion (magenta sphere) with conserved side chains Cys6, Cys9, His24 and His30 (shown as stick models). Other conserved side chains of U1C that were subjected to mutational analysis in Yhc1 are shown as stick models and labeled in regular font. Non-identical side chains that were mutated are denoted in italics: the label specifies the side chain in human U1C, with the yeast Yhc1 side chain in parenthesis. (**B**) Alignment of the primary structures of the N-terminal domains of *S. cerevisiae* (Sce) Yhc1 and human (Hsa) U1C. Positions of side chain identity/similarity are indicated by **•** above the alignment. The α helices are depicted below the alignment as cylinders. The 13 residues of Yhc1 that were subjected to alanine scanning in the present study are highlighted in gold shading. (**C**) Mutational analysis of the zinc binding site. The *C6A*, *C9A*, *H24A* and *H30A* mutants were tested for *yhc1*Δ complementation by plasmid shuffle. *C6A* was lethal. Viable FOA-resistant *C9A*, *H24A* and *H30A* strains were spot-tested for growth on YPD agar at the temperatures specified in parallel with the isogenic wild-type *YHC1* strain.

In the present study, we extend this approach to interrogate structure-function relations and genetic interactions of the essential Yhc1 subunit of yeast U1 snRNP. Yhc1 is a 231-amino acid (aa) polypeptide (Figure 1). Although the N-terminal 40-aa segment of Yhc1 is homologous to the N-terminus of the 159-aa human U1C (27/40 positions of side chain identity or similarity; Figure 2B), the respective C-terminal segments have little or no apparent primary structure similarity (6*,*^38^). By serial deletions, we define the distal margin of a minimal functional Yhc1 protein. We then exploit the NMR structure of the N-terminal domain of human U1C (38) (Figure 2A) to direct mutations to the Cys6-Cys9-His24-His30 zinc binding site and to conserved surface residues that we considered candidates to interact with RNA or protein components of the spliceosome. We identify benign mutations of Yhc1 that display allele-specific synergies with other mutations in the spliceosome, especially the absence of Mud2, Nam8, Mud1, and the TMG cap as well as specific perturbations of the 5′ end of U1 snRNA. We also identify novel Yhc1 mutations that bypass the essentiality of Prp28. We interpret our findings in light of the 5.5 Å crystal structure of the human U1 snRNP that includes the N-terminal domain of U1C (10). Our results add Yhc1 to an extensive reticulum of intramolecular and intermolecular genetic interactions of the U1 snRNP.

## MATERIALS AND METHODS

### Yhc1 expression plasmids and mutants

A 1.7-kb DNA segment bearing the *YHC1* gene was amplified from *S. cerevisiae* genomic DNA by PCR using a forward primer that introduced an XmaI site 490-bp upstream of the Yhc1 translation initiation site and a reverse primer to introduce a NotI site 514-bp downstream of the stop codon. The PCR product was digested with XmaI and Not1 and inserted between the XmaI and NotI sites of centromeric plasmids pRS316 (*URA3*) and pRS413 (*HIS3*) to yield yeast expression plasmids p316-YHC1 and p413-YHC1. Single alanine mutations were introduced into the *YHC1* gene plasmid by two-stage PCR overlap extension with mutagenic primers. The PCR products were digested with XmaI and NotI and inserted into pRS413 to generate a series of p413-YHC1-Ala plasmids. C-terminal truncation variants were generated by PCR using reverse primers that introduced stop codons and a flanking SpeI site in lieu of Asp213, Thr190, Pro171, Gln151, Ser134, Lys117, His88 or Asn66. The truncated PCR products were inserted into pRS413 to generate plasmids p413-YHC1-(1-212), p413-YHC1-(1-189), p413-YHC1-(1-170), p413-YHC1-(1-150), p413-YHC1-(1-133), p413-YHC1-(1-116), p413-YHC1-(1-87) and p413-YHC1-(1-65). The *YHC1* genes were sequenced completely to confirm that no unwanted changes were acquired during amplification and cloning.

### Yeast strains and tests of *YHC1* function *in vivo*

To develop a plasmid shuffle assay for gauging mutational effects on Yhc1 function, we generated a *yhc1*Δ strain that relies for viability on maintenance of the wild-type *YHC1* gene on a *CEN URA3* plasmid, p316-YHC1. In brief, we first replaced the *YHC1* locus from position +1 to +688 with a *hygMX* cassette in the BY4743 diploid strain and then transformed the heterozygous diploid with p316-YHC1. The diploid was sporulated, asci were dissected and haploid *yhc1*Δ Ura^+^ progeny were recovered. The *yhc1*Δ [p316-YHC1] cells were resistant to hygromycin and unable to grow on medium containing 0.75 mg/ml 5-fluoroorotic acid (FOA). To assay the function of wild-type and mutated *YHC1* alleles, *yhc1*Δ [p316-YHC1] cells were transfected with *CEN HIS3 YHC1* plasmids. Individual His^+^ transformants were selected and streaked on agar medium containing FOA. The plates were incubated at 20, 30 or 37°C, and mutants that failed to form macroscopic colonies at any temperatures after 8 d were deemed lethal. Individual FOA-resistant colonies with viable *YHC1* alleles were grown to mid-log phase in YPD broth and adjusted to the same *A*_600_ values. Aliquots (3 µl) of serial 10-fold dilutions were spotted to YPD agar plates, which were then incubated at temperatures ranging from 18 to 37°C.

We also developed plasmid shuffle assays to test the mutational effects on Yhc1 function in *mud2*Δ, *nam8*Δ, *mud1*Δ and *tgs1*Δ cells using standard genetic manipulations of mating, sporulation and dissection. We thereby generated *mud2*Δ *yhc1*Δ [p316-YHC1], *nam8*Δ *yhc1*Δ [p316-YHC1], *mud1*Δ *yhc1*Δ [p316-YHC1] and *tgs1*Δ *yhc1*Δ [p316-YHC1] cells, which were unable to grow on FOA-containing medium unless they had been transformed with wild-type *YHC1* or a functional *YHC1* mutant allele on a *CEN HIS3* plasmid before FOA selection. To investigate genetic interactions of Yhc1 mutants with the essential *U1* and *PRP28* genes, we first generated heterozygous *U1*Δ/*U1 yhc1*Δ/*YHC1* and *prp28*Δ/*PRP28 yhc1*Δ/*YHC1* diploids by crossing *U1Δ*::*kanMX* p316-U1 and *prp28Δ::natMX* p316-PRP28 cells with *yhc1Δ::hygMX* p316-YHC1 cells of the opposite mating type, selecting diploids on YPD medium containing hygromycin plus G418 or hygromycin plus clonNat, and plating them to FOA-containing medium. The heterozygous diploids were then transfected with *CEN URA3 U1 YHC1* (p316-U1-YHC1) or *CEN URA3 PRP28 YHC1* (p316-PRP28-YHC1) plasmids. [In these plasmids, the *YHC1* gene is arranged in a head-to-tail configuration with the *U1* gene (nucleotides −550 to +755) and in a head-to-head configuration with the *PRP28* gene (nucleotides −520 to 2120).] Ura^+^ heterozygous diploids were subjected to sporulation and tetrad dissection, after which haploid *U1*Δ *yhc1*Δ [p316-U1-YHC1] and *prp28*Δ *yhc1Δ* [p316-PRP28-YHC1] progeny were recovered. These cells were unable to grow on FOA medium, but the double-deletion strains could be complemented by co-transformation with p[*CEN HIS3 YHC1*] plus either p[*CEN LEU2 U1*] or p[*CEN LEU2 PRP28*].

We used similar strategies to establish plasmid shuffle assays to test the effects of *SMD3* mutations in *mud1*Δ, *mud2*Δ and *prp28*Δ backgrounds. In the *smd3*Δ [p316-SMD3] haploids used for genetic crosses, the *SMD3* locus (+1 to +303) was replaced by a hygromycin-resistance cassette. The viability of the *smd3*Δ, *smd3*Δ *mud1*Δ and *smd3*Δ *mud2*Δ strains depended on the maintenance of a *CEN URA3* plasmid harboring the *SMD3* gene (nucleotides −445 to +552). The *smd3*Δ *prp28*Δ [p316-PRP28-SMD3] strain harbors a *CEN URA3* plasmid with the *PRP28* and *SMD3* (nucleotides −445 to +552) genes arranged in a head-to-tail configuration.

## RESULTS

### C-terminal truncations define a minimized functional Yhc1 domain

The size differences and lack of amino acid sequence similarity between *S. cerevisiae* Yhc1 and human U1C distal to the N-terminal zinc-binding domain raised the prospect that the yeast and human C-termini might have diverged in parallel with the differences in U1 snRNA size and the number of U1-specific protein subunits of the respective U1 snRNPs. By contrast, an alignment of the 231-aa *S. cerevisiae* Yhc1 to the homologous 189-aa polypeptide from the yeast *Kluyveromyces lactis* highlighted, in addition to the strong conservation of the N-terminus (35/40 positions of side chain identity or similarity), a modest conservation throughout the distal segment of the respective proteins (64/159 positions of side chain identity or similarity), punctuated by two peptide inserts and a 19-aa C-terminal tail unique to *S. cerevisiae* Yhc1 (Figure 1). We used this alignment to guide serial C-terminal truncations of Yhc1, at sites denoted by reverse arrows in Figure 1. The wild-type and truncated alleles were placed on *CEN HIS3* plasmids under the control of the native *YHC1* promoter and tested by plasmid shuffle for complementation of a *yhc1*Δ p[*CEN URA3 YHC1*] strain. The resulting *YHC1-(1-212), YHC1-(1-189)* and *YHC1-(1-170)* strains were viable after FOA selection and grew as well as wild-type *YHC1* cells on YPD agar at all temperatures tested, as gauged by colony size (Figure 1). The *YHC1-(1-150)* strain was viable but sick at all temperatures (Figure 1). The *YHC1-(1-133)*, *YHC1-(1-116)*, *YHC1-(1-87)* and *YHC1-(1-65)* alleles were lethal, i.e. they failed to complement growth of the *yhc1*Δ p[*CEN URA3 YHC1*] test strain on FOA at any temperature tested. We conclude that (i) the C-terminal 61 aa are dispensable for Yhc1 function *in vivo*; (ii) the segment from aa 151-170 is needed for optimal Yhc1 function; and (iii) the segment from aa 134–151 is essential for cell viability.

### Effects of mutating the zinc-binding site of Yhc1

The NMR structure of the N-terminal segment of human U1C (38) revealed a globular fold nucleated by a Cys6-Cys9-His24-His30 zinc coordination complex that is conserved in yeast Yhc1 (Figure 2A and B). Mutating human U1C Cys6 or Cys9 to serine was reported to abolish the binding of U1C to the human U1 snRNP, as did mutating His24 or His30 to glutamine (39). To probe the contributions of the four zinc-binding side chains to yeast Yhc1 activity *in vivo*, we mutated them individually to alanine in the context of full-length Yhc1. The *C6A*, *C9A*, *H24A* and *H30A* alleles were tested by plasmid shuffle for *yhc1*Δ complementation. Although *C6A* was unconditionally lethal, we were surprised to find that *C9A*, *H24A* and *H30A* cells were viable. The *H24A* strain was relatively healthy, growing on YPD agar at all temperatures with colony size slightly smaller than wild-type (Figure 2C). The *C9A* and *H30A* strains grew well at low temperatures (18–20°C) but did not thrive at high temperatures (30–37°C) (Figure 2C). These results show that Yhc1 function *in vivo* can be sustained when Cys6 and any two of the other three zinc-binding residues are present. It is conceivable that, when Cys9, His24 or His30 are replaced by alanine, a water can substitute for the missing amino acid sulfur or nitrogen atom in the tetrahedral zinc coordination complex, albeit with some loss of stability/activity, as reflected in the slower growth or temperature sensitive (ts) growth of the respective alanine mutants.

### Effects of alanine mutations of conserved surface residues in the N-terminal module

The N-terminal domain of Yhc1/U1C is imputed to make physical contacts with the 5′ leader sequence of U1 snRNA, the pre-mRNA sequence in the vicinity of the 5′SS, and with other protein components of the U1 snRNP (6*,*^10^*,*^12^*,*^40^*,*^41^*,*^42^). In our view, the likely candidates to mediate these RNA and protein contacts are to be found within the set of conserved basic and hydrophilic side chains that emanate from the surface of the N-terminal domain (Figure 2A). Therefore, we targeted nine surface-exposed residues for alanine scanning: Arg3, Tyr12, Thr14, His15, Arg21, Lys22, Asn29, Lys28 and Asp36 (Figure 2A and B). The *YHC1-Ala* alleles were tested for *yhc1*Δ complementation by plasmid shuffle and all of them were found to retain biological activity; each of the *YHC1-Ala* strains grew as well as wild-type *YHC1* on YPD agar at all temperatures tested (Supplementary Figure S1).

### Synthetic genetic interactions of Yhc1-Ala mutants with Mud2, Nam8, Mud1 and Tgs1

**Figure 3.**
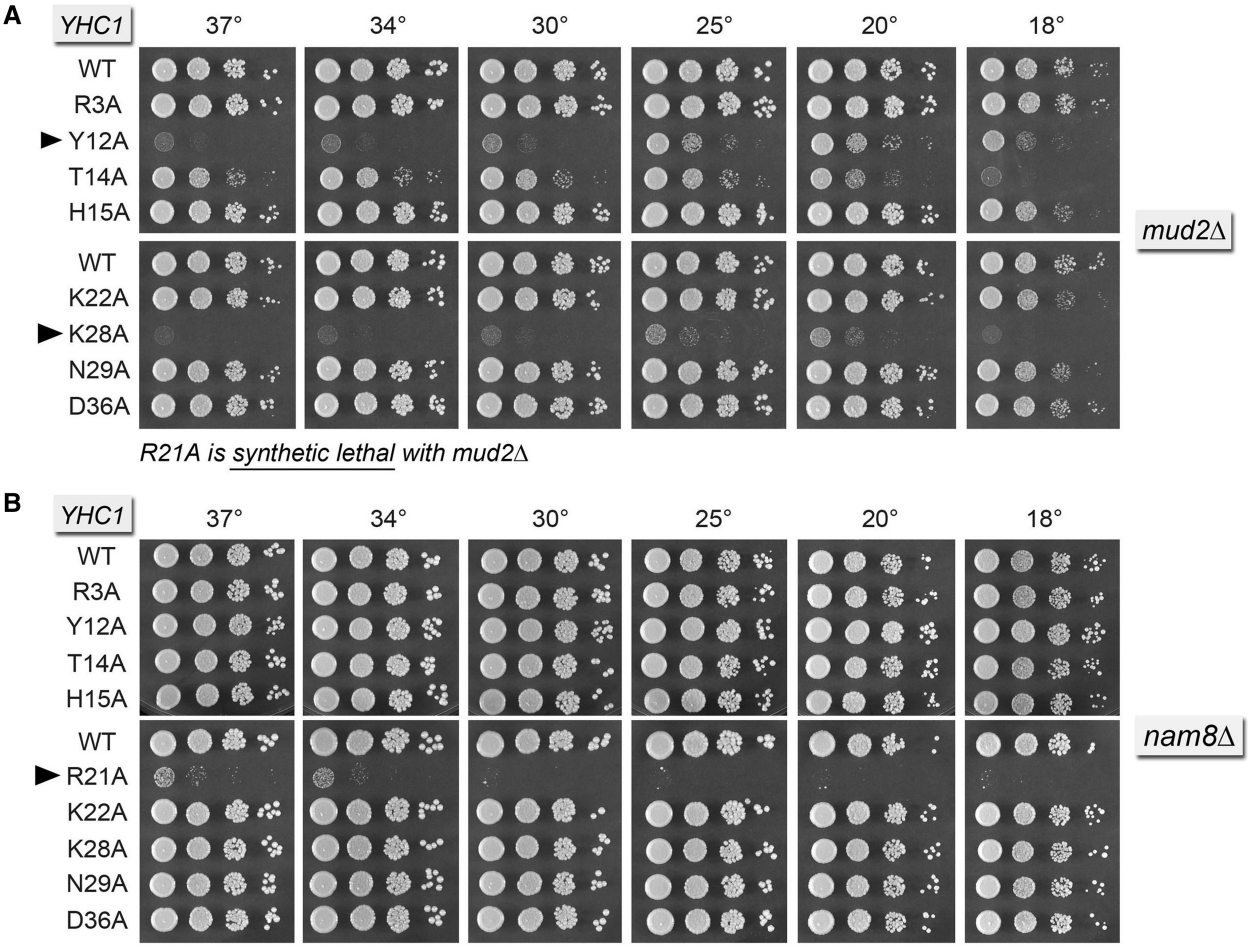
Synthetic interactions of Yhc1-Ala mutants with Mud2 and Nam8. Yeast strains bearing the indicated *YHC1* alleles in the *mud2*Δ (panel A) or *nam8*Δ (panel B) backgrounds were spot-tested for growth at the temperatures specified. *YHC1-R21A* failed to complement *mud2*Δ *yhc1*Δ in a plasmid shuffle assay and was deemed synthetic lethal. Alleles with strong synthetic growth defects are indicated by ► at *left*.

To survey genetic interactions of the apparently fully active Yhc1-Ala mutants, we constructed a series of yeast strains in which the genes encoding inessential splicing factors Mud2, Nam8 or Mud1 were deleted in the *yhc1*Δ p[*CEN URA3 YHC1*] background, thereby allowing tests of synergy by plasmid shuffle. Although the ablation of Mud2 has no effect on yeast vegetative growth by itself, *mud2*Δ caused synthetic lethality in combination with the *YHC1-R21A* allele (Figure 3A). *mud2*Δ elicited strong ‘synthetic sick’ phenotypes in combination with *YHC1* mutants *K28A* and *Y12A*, manifested as no growth at 30–37°C and minimal growth at ≤25°C (Figure 3A). *YHC1-T14A* had a cold-sensitive (cs) growth defect in the *mud2*Δ background. The *YHC1* mutants *R3A*, *H15A*, *K22A*, *N29A* and *D36A* displayed no synergy with *mud2*Δ.

Loss of U1 snRNP subunit Nam8 has no effect on yeast vegetative growth, yet *nam8*Δ caused a strong synthetic growth defect in combination with *YHC1* mutant *R21A* allele, whereby the *YHC1-R21A nam8*Δ strain formed pinpoint colonies at 37 and 34°C and failed to grow at ≤30°C. By contrast, none of the other eight *YHC1-Ala* mutants displayed a growth defect in the *nam8*Δ background (Figure 3B).

Two of the *YHC1-Ala* mutants had conditional synthetic defects in a yeast strain that lacked the Mud1 subunit of the U1 snRNP. *YHC1-R21A mud1*Δ cells were ts at 37°C and cs at 18°C. *YHC1-Y12A* cells were ts at 37°C. The seven other Ala mutants grew normally in the *mud1*Δ background (Supplementary Figure S2).

**Figure 4.**
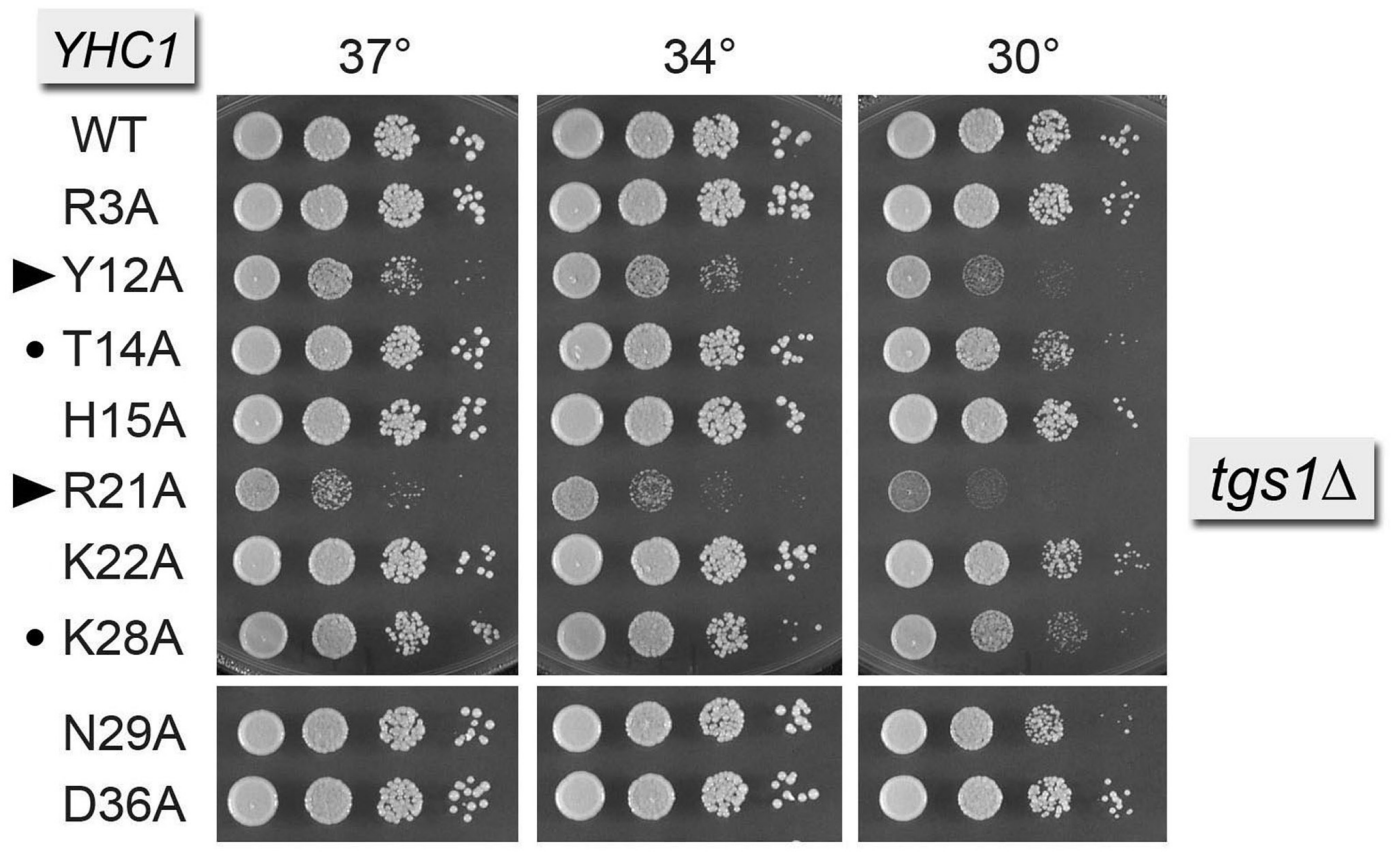
Synthetic interactions of Yhc1-Ala mutants with Tgs1. Yeast *tgs1*Δ *yhc1*Δ strains bearing the indicated *YHC1* alleles were spot-tested for growth at the temperatures specified. The stronger synthetic growth defects are indicated by ► at *left*. Alleles with milder synergies are denoted by •.

Tgs1 is the enzyme responsible for conversion of m^7^G RNA caps to the m^2,2,7^G trimethylguanosine caps that are a signature feature of the U1, U2, U4 and U5 snRNAs (17*,*^43^*,*^44^). Yeast *tgs1*Δ cells are viable and grow well at 30–37°C, but they fail to grow at 18–20°C. Here, we constructed a *tgs1*Δ *yhc1Δ* p[*CEN URA3 YHC1*] strain to test synergies between the absence of a TMG cap and the Yhc1-Ala mutants. We found that the *YHC1-R21A tgs1*Δ strain was very sick, forming pinpoint colonies at 37 and 34°C and failing to thrive at 30°C (Figure 4). The *YHC1-Y12A tgs1*Δ strain had a similar strong synthetic phenotype (Figure 4). The *T14A* and *K28A* alleles elicited mild synthetic effects in the *tgs1*Δ background (Figure 4).

### Synthetic genetic interactions of Yhc1-Ala mutants with alterations of the U1 snRNA

**Figure 5.**
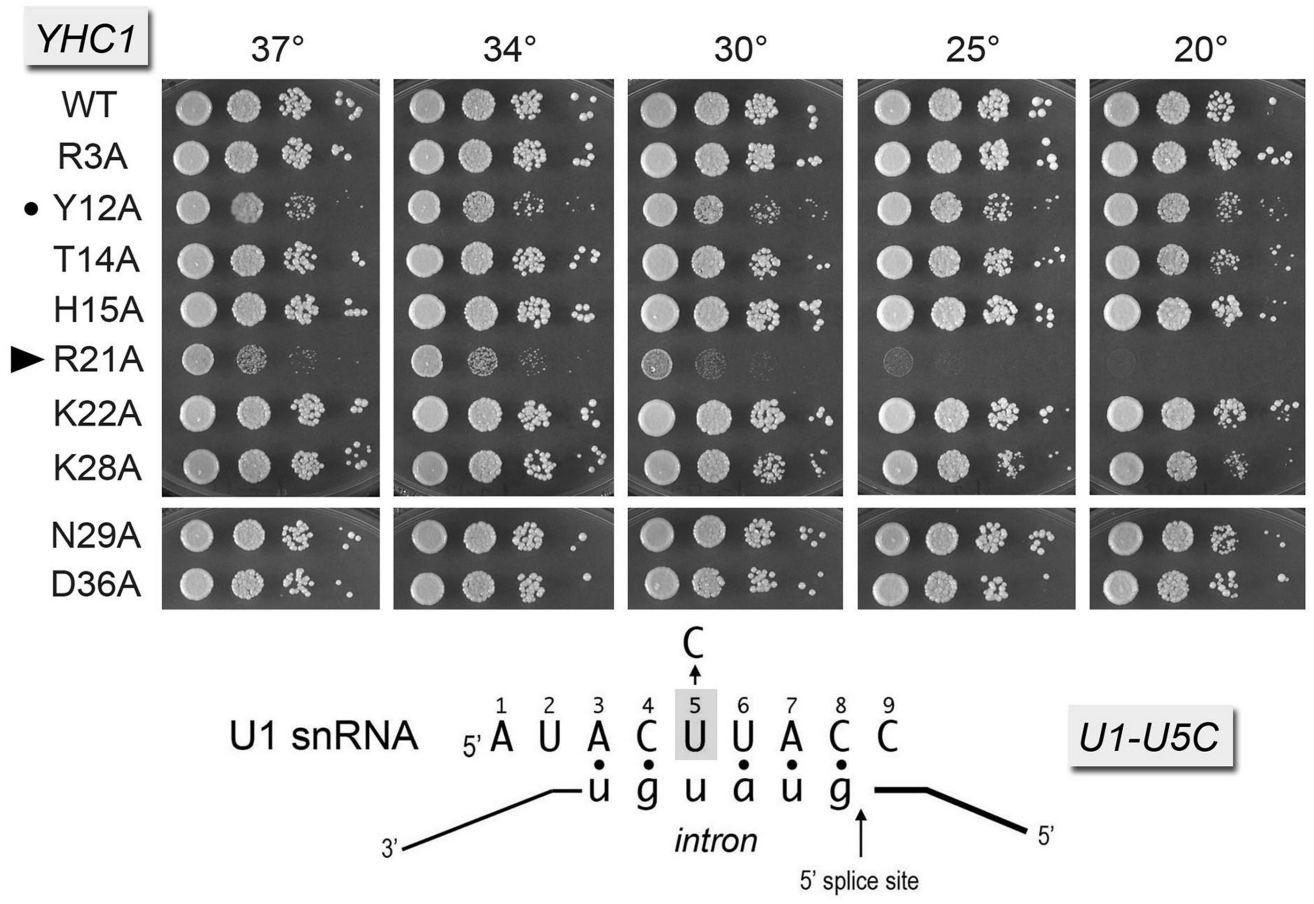
Synthetic interactions of Yhc1-Ala mutants with U1 snRNA mutant U^5^C. The *U1-U^5^C yhc1*Δ strains bearing the indicated *YHC1* alleles were spot-tested for growth at the temperatures specified. The severe synthetic growth defect of the *R21A* allele is denoted by ► at *left*. The milder synergy with *Y12A* is denoted by •.

The base-pairing interaction between the 5′ leader sequence ^3^ACUUAC^8^ of yeast U1 snRNA and the consensus 5′SS of yeast pre-mRNAs is depicted in Figure 5. Recently, we reported that mutating U1 snRNA nucleobase U^5^ to C had no effect on yeast vegetative growth, signifying that a Py^5^:U mispair in the U1•5′SS RNA hybrid suffices for seemingly normal U1 snRNA activity (25). Yet, we observed strong genetic interactions of *U1-U^5^C* with *mud2*Δ (unconditional synthetic lethality) and *nam8*Δ (slow growth at 37 and 34°C as well as failure to grow at 30, 25 and 20°C) (25). Here we tested for genetic interactions of U1-U^5^C with the Yhc1-Ala mutants, by constructing a *U1*Δ *yhc1*Δ p[*CEN URA3 U1 YHC1*] strain, co-transforming it with *YHC1-Ala* and *U1-U^5^C* plasmids, screening for growth on FOA and spot testing the FOA-resistant *YHC1-Ala U1-U^5^C* strains for growth on YPD agar (Figure 5). The *YHC1-R21A U1-U^5^C* strain was uniquely sick, forming pinpoint colonies at 37, 34 and 30°C, and failing to thrive at 25 and 20°C. The *YHC1-Y12A U1-U^5^C* strain displayed a mild slow growth phenotype at all temperatures.

Extending the 5′ leader sequence by adding 25 nt upstream of the native 5′ end (as depicted in Supplementary Figure S3) had no effect *per se* on yeast vegetative growth. However, the *U1+25* allele was synthetic lethal with *mud2*Δ and synthetic sick with *nam8*Δ and *tgs1*Δ (25). Here we examined the effects of otherwise benign Yhc1-Ala mutants in the *U1+25* background. Again, the *YHC1-R21A* allele was uniquely synthetically sick with *U1+25* (Supplementary Figure S3).

**Figure 6.**
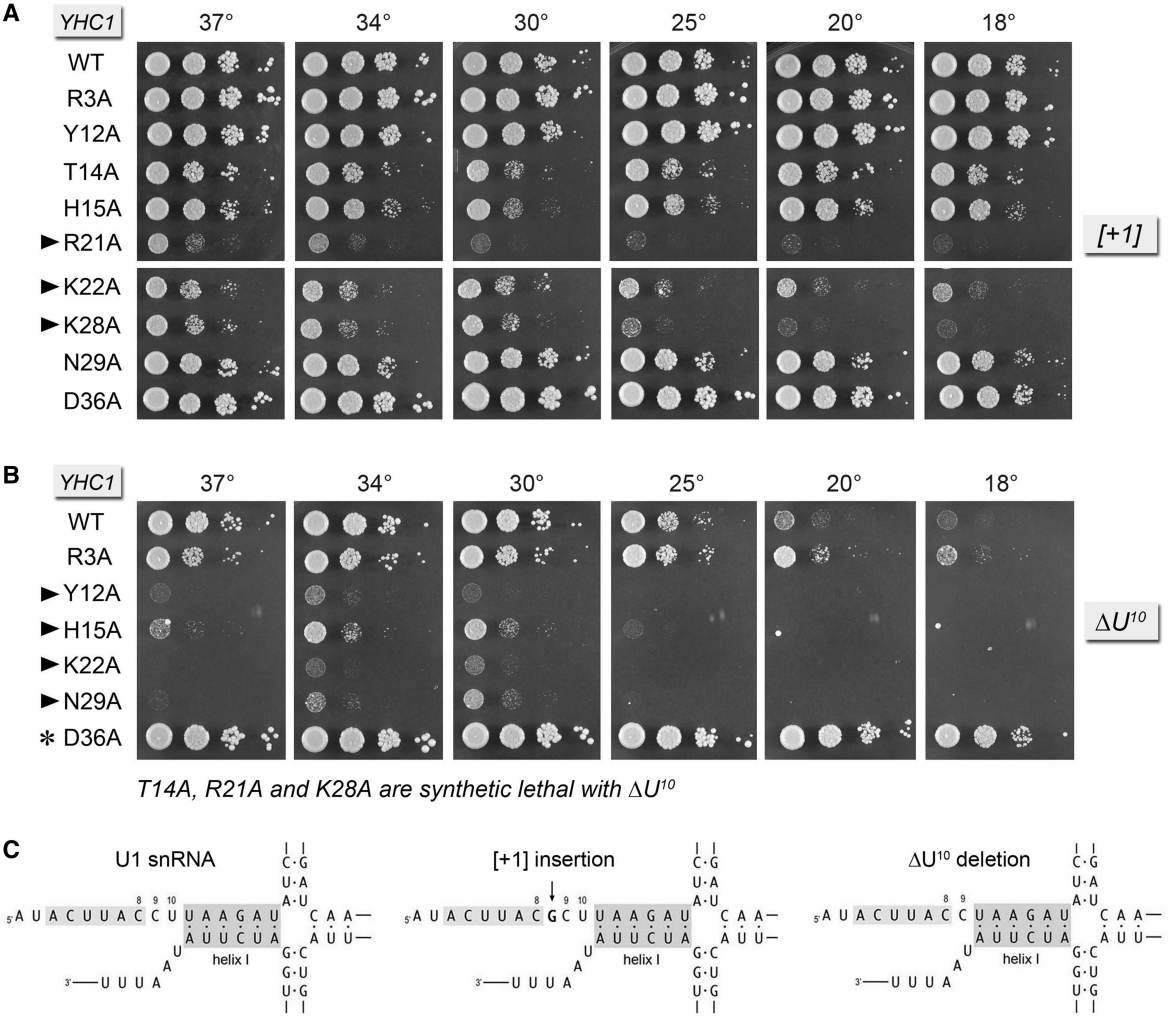
Genetic interactions of Yhc1-Ala mutants with U1 snRNA mutants *[+1]* and *ΔU^10^*. Yeast strains bearing the indicated *YHC1* alleles in the *U1-[+1]* (panel A) or *U1-ΔU^10^* (panel B) backgrounds were spot-tested for growth at the temperatures specified. *YHC1* alleles that failed to complement *U1-ΔU^10^ yhc1*Δ in a plasmid shuffle assay and were deemed synthetic lethal are indicated below panel B. Alleles with strong synthetic growth defects are indicated by ► at *left*. Suppression of the *U1-ΔU^10^* growth defect by *YHC1-D36A* is denoted by an asterisk at *left*. (Panel C) The primary and predicted secondary structures at the 5′ end of wild-type U1 RNA and the *[+1]* insertion and *ΔU^10^* deletion mutants are shown. The ^3^ACUUAC^8^ segment that pairs with the intron 5′SS is highlighted in light gray. The paired bases that comprise helix 1 are highlighted in dark gray.

The 5′ AUACUUACCU^10^ single-stranded segment of U1 snRNA precedes the folded U1 RNA tertiary structure that initiates at nucleotide U^11^ (i.e. a four-helix junction depicted in Figure 6C). The ^8^CCU^10^ segment connecting the 5′SS complementary motif to helix 1 interacts with the U1C subunit in the U1 snRNP (6*,*^10^). We showed previously that the spacing between the 5′SS complementary motif and helix 1 is important, insofar as insertions of 5 or more nucleotides between C^8^ and C^9^ of yeast U1 snRNA were uniformly lethal (25). By contrast, insertion of a single G nucleotide, as in the *[+1]* allele in Figure 6C, was viable. However, the *[+1]* insertion was lethal in combination with *nam8*Δ, *mud1*Δ, *tgs1*Δ and *mud2*Δ (25). Here we found that the Yhc1 mutants *R21A*, *K22A* and *K28A* displayed severe synthetic sick phenotypes in a *U1-[+1]* background (Figure 6A). The *T14A U1-[+1]* and *H15A U1-[+1]* strains had a milder synthetic defect, growing slowly at 25–34°C (Figure 6A).

Helix 1 is conserved with respect to its presence and position in yeast and human U1 snRNAs. To probe the effects of increasing the length of yeast helix 1, the ^9^CU^10^ dinucleotide was changed to ^9^UA^10^, thereby extending the base-pairing potential to the sequence at the 3′ end of U1 RNA, such that helix 1 might span 8 bp instead of 6 bp (Supplementary Figure S4). This *U1* allele, named *H1L* (‘helix 1 long’), supported normal yeast growth but was synthetic lethal with *mud2*Δ and synthetic sick with *nam*8Δ, *mud1*Δ and *tgs1*Δ (25). Of the collection of Yhc1-Ala mutants, *R21A* was uniquely synthetic sick in the *U1-H1L* background (Supplementary Figure S4).

The strongest genetic interactions of the Yhc1-Ala mutants with U1 snRNA were seen when the spacing between the 5′SS complementary motif and helix 1 was shortened by deletion of the U^10^ nucleotide, as depicted in Figure 6C. The *U1-ΔU^10^* single mutant grows normally at 30–34°C but does not thrive at cold temperatures (Figure 6B). We reported previously that *U1-ΔU^10^* was synthetic lethal with *nam8*Δ, *mud1*Δ, *tgs1*Δ and *mud2*Δ (25). Here, we found that Yhc1 mutants *T14A*, *R21A* and *K28A* were synthetic lethal with *U1-ΔU^10^*, whereas *Y12A*, *H15A*, *K22A* and *N29A* displayed severe synthetic sickness in the *U1-ΔU^10^* background (Figure 6B). However, on the contrary, the *YHC1-D36A* allele elicited a gain-of-function effect in the *U1-ΔU^10^* background, fully suppressing the *U1-ΔU^10^* cs growth defect (Figure 6C, compare WT and D36A).

### Bypass of the essentiality of Prp28 by Yhc1 R21A and K28A mutations

Yeast Prp28 is an essential pre-mRNA splicing factor and a member of the DEAD-box family of nucleic acid-dependent NTPases (45*,*^46^). Prp28 is implicated genetically in displacing the U1 snRNP from the 5′SS during the transition from a pre-mRNA•U1•U2-containing spliceosome to a pre-mRNA•U2•U4•U6 spliceosome (4*,*^5^,^47^). The strongest evidence for this idea emerges from the findings that the essentiality of Prp28 for vegetative growth can be bypassed by mutations in the essential U1 snRNP subunits Yhc1, Prp42 and Snu71, and by specific U1 snRNA mutations located within and flanking the segment that base pairs with the intron 5′SS (4*,*^25^*,*^47^). These results highlight that the need for Prp28 during U1 snRNP ejection from the early spliceosome is alleviated by certain alterations that are presumed to weaken U1•5′SS contacts.

Yhc1 figures prominently in the U1 snRNP-Prp28 dynamic, insofar as Chen *et al.* (4) identified in a genetic screen two independent mutations of the same amino acid in Yhc1 (Leu13 changed to either Phe or Ser) that allowed growth of a *prp28*Δ strain. They proceeded to test all 19 possible substitutions at Leu13 and found that (i) any amino acid at position 13 sufficed for complementation of *yhc1*Δ and (ii) 13 of the amino acids allowed some level of growth of *prp28*Δ (4). They proposed that the conserved Leu13 residue ‘makes specific contact’ to stabilize the U1•5′SS duplex (4). The subsequent report of the NMR structure of human U1C (38) revealed that the Leu13 side chain points into the globular core of the N-terminal zinc-binding domain, where it makes van der Waals contacts via Cδ2 to the His24 side chain that coordinates the zinc ion (Figure 2A). The structure indicates that Leu13 is not in a position to interact with another protein or RNA, making it likely that the Prp28 bypass elicited by Leu13 mutations is an indirect effect of a conformational change in the Yhc1 N-terminal domain.

**Figure 7.**
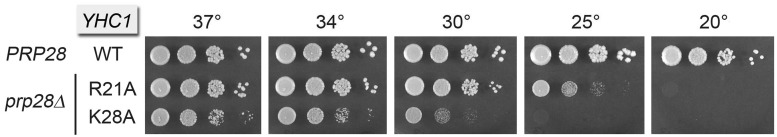
Yhc1 R21A and K28A bypass the requirement for the essential *PRP28* gene. Yeast *prp28*Δ *yhc1Δ* cells harboring the indicated *YHC1* allele on a *CEN HIS3* plasmid and either wild-type *PRP28* (*CEN LEU2*) or an empty *CEN LEU2* plasmid (*prp28*Δ) were grown in liquid cultures at 37°C to mid-log phase. The cultures were adjusted to *A*_600_ of 0.1, and aliquots of serial dilutions were spotted to YPD agar. Photographs of the plates after incubation for 2 d (30, 34, 37°C) or 4 d (20, 25°C) are shown.

We envisioned a scenario in which subtraction of surface-exposed side chains of the Yhc1 N-terminal domain might weaken the U1•5′SS contacts and thereby bypass the requirement for Prp28. To put this idea to the test, we constructed a *yhc1*Δ *prp28*Δ p[*CEN URA3 PRP28 YHC1*] strain, transfected it with wild-type *YHC1* and each of the nine *YHC1-Ala* plasmids and then screened the transformants for growth on FOA, i.e. survival in the absence of *PRP28*. Two *YHC1* mutant alleles passed this test: *R21A* and *K28A*. The viable *yhc1*Δ *prp28*Δ cells bearing these bypass alleles were tested for growth on YPD agar at 20, 25, 30, 34 and 37°C (Figure 7). *R21A* was the better of the Prp28 bypass suppressors, insofar as *YHC1-R21A prp28*Δ cells grew about as well as wild-type cells at 37, 34 and 30°C, as gauged by colony size (Figure 7). That they grew slowly at 25°C and did not thrive at 20°C signified that the *R21A* bypass effect is cs (Figure 7). This result suggests that higher temperatures help destabilize the weakened U1•5′SS pairing in *YHC1-R21A* cells and thereby permit U1 snRNP ejection without the assistance of Prp28. However, when the pairing is more stable at lower temperatures, Prp28 is still required. The *YHC1-K28A* allele allowed Prp28 bypass at 37 and 34°C, but was less effective at 30°C and ineffective at 25°C (Figure 7).

### Interactions of Yhc1 Arg21 with the SmD3 subunit of the Sm ring

**Figure 8.**
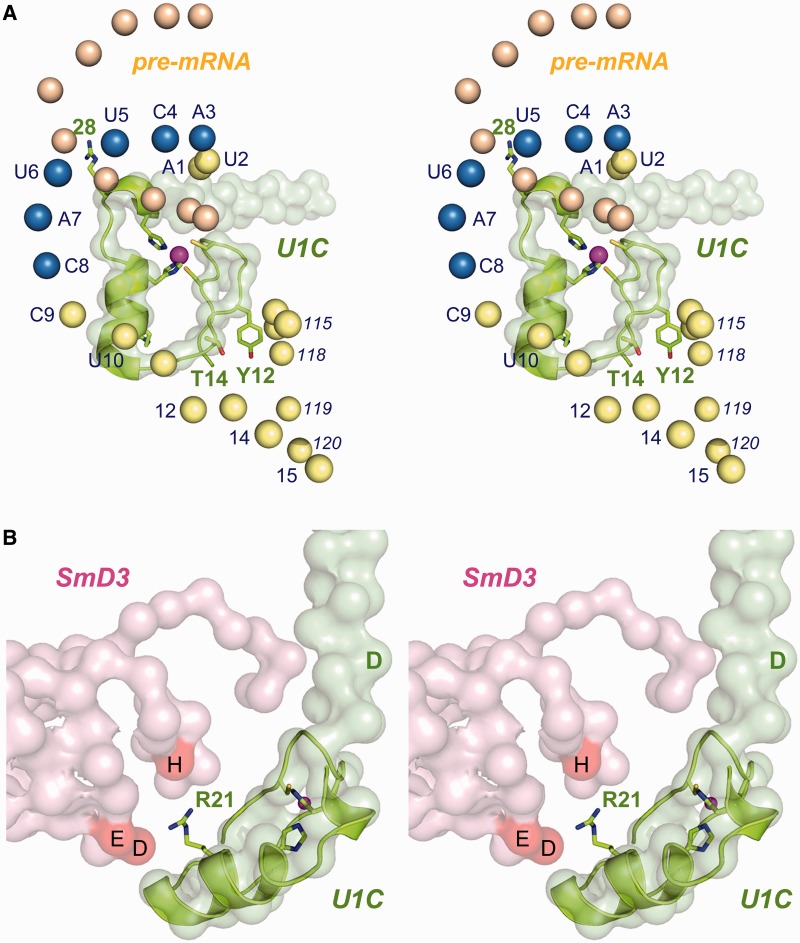
Disposition of U1C in the structure of the U1 snRNP. (**A**) Stereo view of the U1C N-terminal domain (light green surface model) and a nearby segment of the U1 snRNA from the 5.5 Å crystal structure of the human U1 snRNP (pdb 3CW1). The NMR structure of U1C-(1-31) (from pdb 2VRD) is superimposed on the crystal structure, with selected side chain depicted as stick models and the Zn^2+^ ion as a magenta sphere. The phosphate atoms of the U1 snRNA are shown as spheres with the nucleotide numbers specified nearby; the ^3^ACUUAC^8^ motif complementary to the 5′SS is in blue. The U1 5′ end base-pairs with its counterpart from an adjacent U1 snRNP complex in the crystal, mimicking the pairing of U1 with nucleotides at the 5′SS of the pre-mRNA intron (colored beige). (**B**) Stereo view of the U1C (green surface model) and SmD3 (pink surface model) subunits of human U1snRNP, with superimposed NMR structure of U1C, highlighting the proximity of Arg21 (green stick model) to SmD3 residues His11 (H), Glu36 (E) and Asp37 (D). The U1C Asp36 residue is indicated by the green letter D.

Inspection of the low-resolution U1 snRNP structure containing the U1C N-terminal domain (with the higher-resolution U1C fold superimposed) indicates that the Yhc1/U1C Arg21 residue is disposed on the protein surface opposite from the face that contacts the intron 5′SS or the 5′ segment of U1 snRNA (Figure 8A). Rather, the view in Figure 8B reveals that Arg21 is a likely component of the interface of Yhc1/U1C with the SmD3 subunit of the Sm protein ring. Although atomic contacts cannot be surmised from the structures, the proximity of the basic Arg21 residue to human SmD3 residues His11, Glu36 and Asp37 (H, E and D in Figure 8B) hints that Arg21 might participate in ionic and/or hydrogen bonding interactions with the corresponding side chains (Asn12, Glu37 and Asp38 in yeast SmD3) at the putative Yhc1-SmD3 interface of the yeast U1 snRNP. Thus, we speculated that perturbing the Yhc1-SmD3 interaction accounts for the widespread mutational synergies of *YHC1-R21A*, whereby splicing depends on Mud2, Mud1, the TMG cap and so forth, because *R21A* is a hypomorph that affects the formation and/or stability of the U1•pre-mRNA•[Msl5•Mud2] early spliceosome intermediate. This view is consistent with our finding that *R21A* bypasses the lethality of *prp28*Δ, presumably because the R21A mutation destabilizes the U1•5′SS complex in the later spliceosome intermediate (pre-mRNA•U1•U2•U5•U4•U6) that is the putative substrate for Prp28-mediated U1 snRNP ejection.

**Figure 9.**
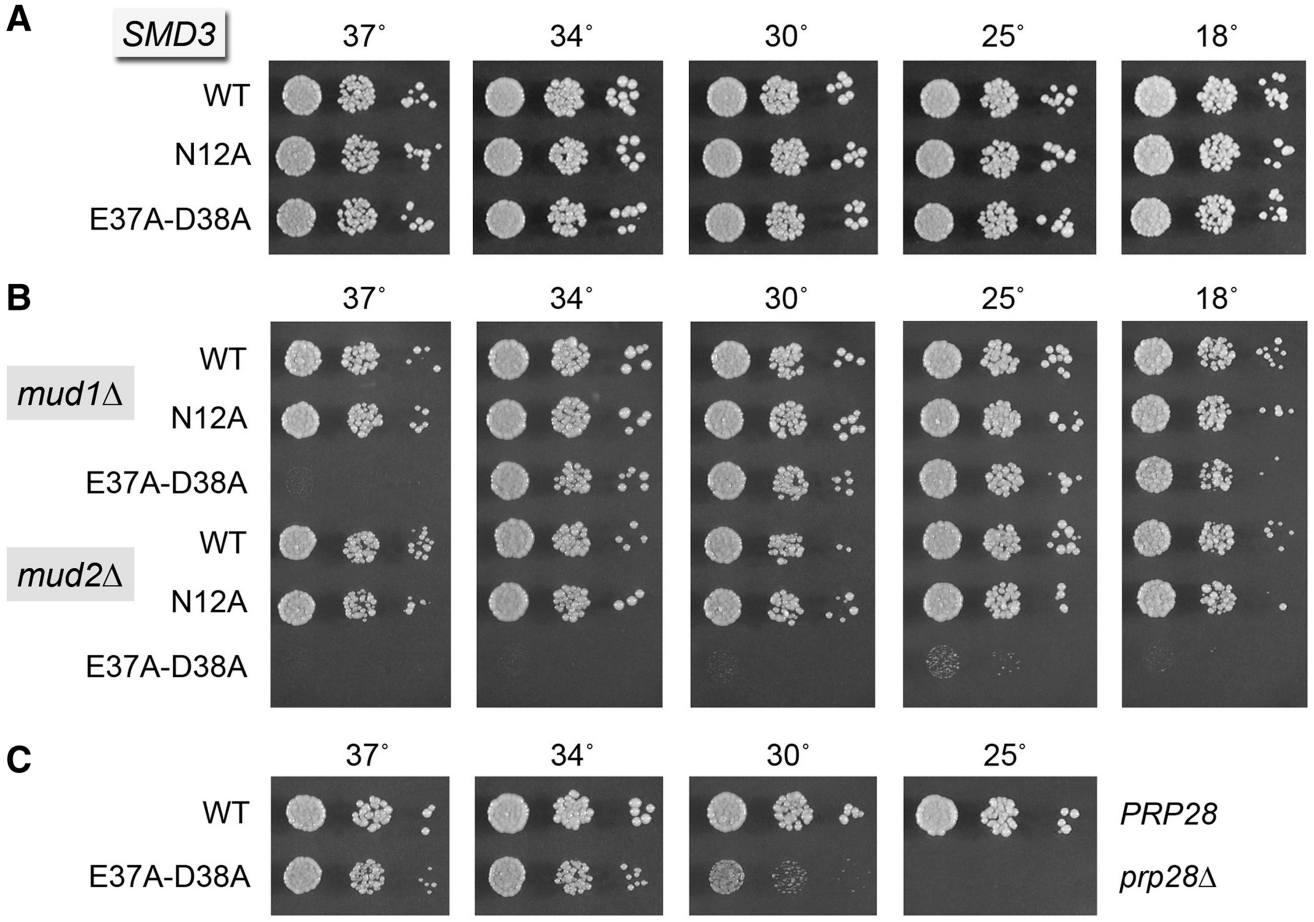
Genetic interactions of SmD3 mutants. (**A** and **B**) Mutational synergies with *mud1*Δ and *mud2*Δ. Yeast *smd3*Δ strains bearing the indicated *SMD3* allele on *CEN LEU2* plasmid in an otherwise wild-type (panel A), *mud1*Δ (panel B) or *mud2*Δ (panel B) background were spot-tested for growth on YPD agar at the temperatures specified. (**C**) Bypass of *prp28*Δ. Yeast *smd3*Δ *prp28*Δ cells bearing wild-type *SMD3* or *SMD3-E37A-D38A* on a *CEN LEU2* plasmid and either p(*CEN HIS3 PRP28*) or an empty *CEN HIS3* plasmid were grown in liquid culture at 37°C. After adjustment to the same cell density (by *A*_600_), serial 10-fold dilutions were spotted to YPD agar. The plates were photographed after incubation for 2 d at 30, 34 and 37°C or 3 d at 25°C.

To evaluate this scenario, we established a plasmid shuffle assay to gauge the effects of mutations N12A and E37A-D38A in the putative interface of yeast SmD3 with Yhc1 Arg21. *smd3*Δ cells bearing *SMD3-N12A* and *SMD3-E37A-D38A* plasmids were viable and grew as well as *smd3*Δ *SMD3* cells on YPD agar at all temperatures tested (Figure 9A). We then queried the *SMD3* alleles for their ability to complement *smd3*Δ *mud2*Δ and *smd3*Δ *mud1*Δ strains. Although no synergies were evident for *SMD3-N12A*, the *E37A-D38A* allele was severely synthetically sick in the absence of Mud2, resulting in only microscopic growth on YPD agar at 25°C and 18°C and no growth at higher temperatures (Figure 9B). Also, the *E37A-D38A* allele elicited a tight ts growth defect at 37°C in the *mud1*Δ background (Figure 9B). Thus, loss of the SmD3 Glu37 and Asp38 moieties, one or both of which is poised to make a salt bridge to Yhc1 Arg21, recapitulated several of the synthetic genetic interactions described for *R21A*, fortifying our hypothesis that the SmD3-Yhc1 interface is pertinent to the genetics of Yhc1 Arg21.

As a further test of this model, we asked whether the *SMD3-E37A-D38A* allele could bypass *prp28*Δ and found that it did, allowing for growth of *SMD3-E37A-D38A prp28*Δ cells at 37 and 34°C (Figure 9C). The *SMD3-E37A-D38A prp28*Δ strain grew slowly at 30°C and did not thrive at ≤25°C, reaffirming that *prp28*Δ bypass mutants are generally cs. The *SMD3-N12A* allele did not bypass *prp28*Δ. We infer that contacts between Yhc1 Arg21 and SmD3 Glu37 and/or Asp38 stabilize the U1 snRNP•5′SS complex.

### Yhc1-D36A exacerbates cs Prp28 mutations

**Figure 10.**
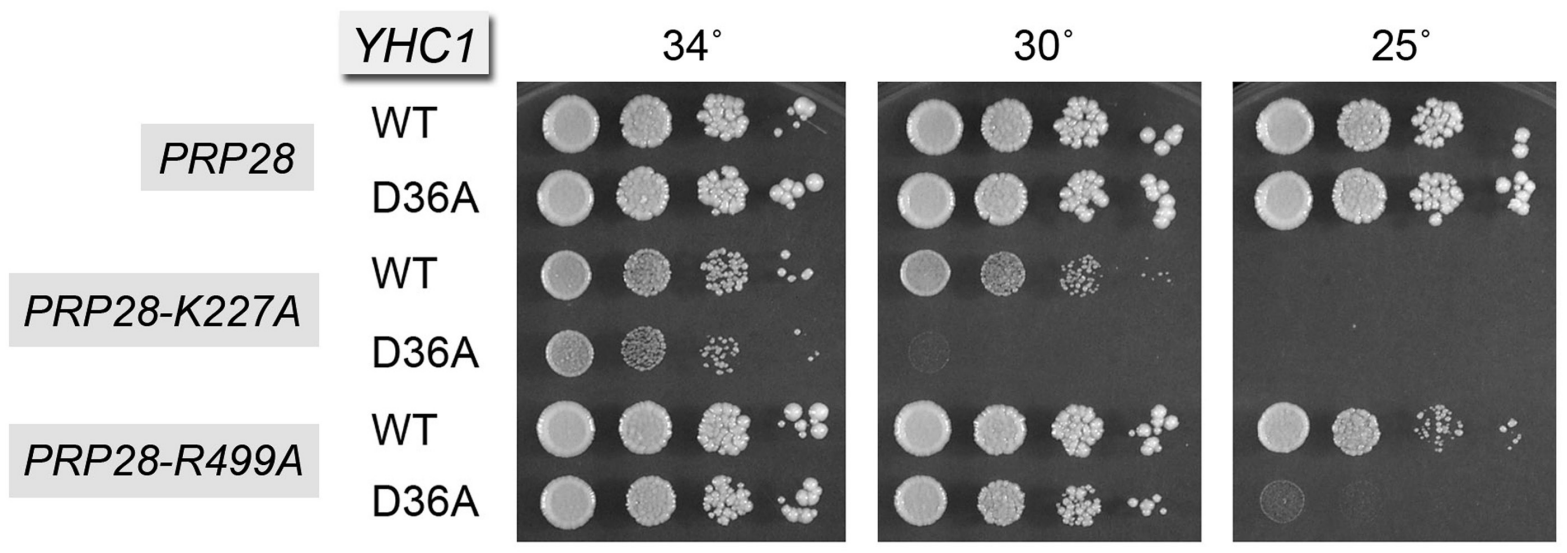
Yhc1-D36A exacerbates cs Prp28 mutations. Cultures of *yhc1*Δ *prp28*Δ cells bearing the indicated *PRP28* and *YHC1* alleles on *CEN HIS3* or *CEN LEU2* plasmids, respectively, were grown at 34°C and spot-tested for growth on YPD agar. The plates were photographed after incubation for 3 d at 30 and 34°C or 4 d at 25°C.

The finding that the *YHC1-D36A* allele suppressed the *cs* growth defect of *U1-ΔU^10^* (Figure 6C) suggested that the D36A change might stabilize interactions of the U1 snRNA or snRNP with the 5′SS. If this is the case, the stability-enhancing *YHC1-D36A* allele ought to synergize with cs mutants of Prp28, which are presumed to be defective in dissociating the U1 snRNP from the spliceosome at low temperatures, when the U1•5′SS base-pairing interactions are more stable, but which can function at higher temperatures that destabilize the U1•5′SS duplex. As a part of a separate effort to chart the structure-function relations of yeast Prp28 by alanine scanning, we identified two mutations that conferred cs growth: K227A and R499A. Lys227 is located within the Prp28 P-loop motif (GSGKT) and is imputed to contact the ATP β- and γ-phosphates. Arg499 is predicted, based on the crystal structure of the homologous DEAD-box protein Vasa (48), to make salt bridges to the Glu342 and Asp344 side chains of the Prp28 DEAD-box. The restrictive temperatures for the *PRP28 K227A* and *R499A* strains were 25 and 20°C, respectively (Figure 10 and data not shown). However, in the *YHC1-D36A* background, the restrictive temperatures were shifted upward, such that *YHC1-D36A PRP28-K227A* cells did not grow at 30°C and *YHC1-D36A PRP28-R499A* cells did not thrive at 25°C (Figure 10). These results are consistent with the idea that Yhc1-D36A enhances U1•5′SS stability. Asp36 is located within the long α helix that follows the globular N-terminal domain of the U1C protein (Figure 8B). Interactions of Asp36 cannot be surmised from the structure.

### Genetic interactions of Yhc1 C-terminal truncations

**Figure 11.**
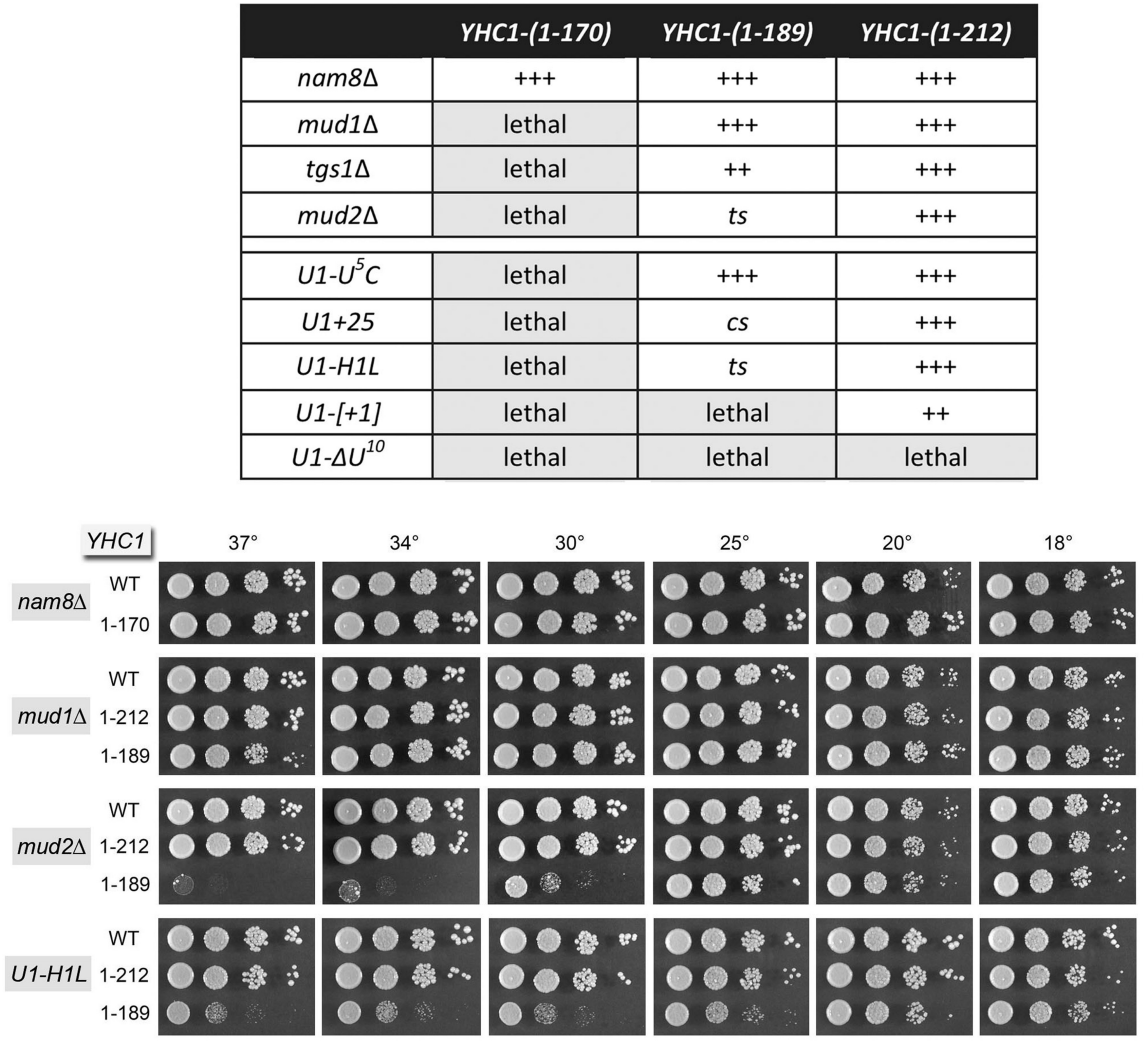
Synthetic genetic interactions of Yhc1 C-terminal truncations. *(Top panel)* Plasmid shuffle assays were used to test whether the truncated *YHC1* alleles were functional in the genetic backgrounds specified in the left column. Synthetic lethality was indicated by failure to form colonies on FOA agar after 8 d at 20, 30 and 37°C. Cultures of viable FOA-resistant cells were grown in YPD broth at 30°C and the growth phenotypes were assessed by spotting serial 10-fold dilutions. Exemplary spot tests are shown in the *bottom panel*. Growth is tabulated in the *top panel* as follows: +++ denotes growth similar to the pertinent strains bearing wild-type *YHC1*; ++ indicates smaller colony size than the strain bearing wild-type *YHC1*; ts signifies a temperature-sensitive growth defect; cs indicates a cold-sensitive growth defect.

In light of the allele-specific mutational synergies involving the N-terminal domain of Yhc1, we proceeded to survey genetic interactions of the benign Yhc1 C-terminal truncations. The results (compiled in Figure 11, *top panel*) disclosed an informative hierarchy of synthetic mutational effects. *YHC1-(1-212)* was uniquely lethal in combination with U1 snRNA mutant *ΔU^10^* but had no effect on growth in combination with *mud1*Δ, *tgs1*Δ or *mud2*Δ or with U1 snRNA mutants *U^5^C*, *+25* or *H1L.* The *YHC1-(1-212) U1-[+1]* strain displayed a mild synthetic defect compared with *YHC1 U1-[+1]* (scored as ++ growth). We conclude that the otherwise dispensable C-terminal segment of Yhc1 from aa 213 to 231 becomes essential under a very specific genetic circumstance.

At the other extreme, the *YHC1-(1-170)* allele encoding the shortest of the biologically active Yhc1 proteins was lethal in combination with *mud1*Δ, *tgs1*Δ and *mud2*Δ and with U1 snRNA mutants *U^5^C*, *+25*, *H1L*, *[+1]* and *ΔU^10^* (Figure 11). Yet, *YHC1-(1-170)* was entirely benign in the *nam8*Δ background (+++ growth; see Figure 11, *bottom panel*). This result diverged sharply from the strong synergy of *nam8*Δ with *YHC1-R21A* (Figure 3B) and suggested that the N and C domains of Yhc1 have distinctly buffered functions during pre-mRNA splicing.

The intermediate truncation allele *YHC1-(1-189)* displayed a spectrum of mutational synergies in different genetic backgrounds. Because *YHC1-(1-189) mud1*Δ cells grew about as well as *YHC1 mud1*Δ cells at all temperatures tested (Figure 11, *bottom panel*), whereas *YHC1-(1-170) mud1*Δ cells were inviable, we surmise that the Yhc1 segment from aa 171 to 189 is genetically redundant with the Mud1 subunit of the U1 snRNP. We found that *YHC1-(1-189) mud2*Δ cells grew well at lower temperatures (18–25°C) but were tightly ts and failed to thrive at 34–37°C (Figure 11, *bottom panel*). In comparison with the lethality of *mud2*Δ to *YHC1-(170)* and the normal growth of *YHC1 mud2*Δ cells, this result signifies that the Yhc1 segment from aa 171 to 189 compensates for the absence of Mud2 at low temperatures, whereas the Yhc1 segment from aa 190 to 212 is required to buffer Mud2 loss at high temperatures. The *YHC1-(1-189) tgs1*Δ strain displayed a mild synthetic defect compared with the *YHC1 tgs1*Δ, seen as smaller colony size at 34–37°C (not shown) and scored as ++ growth (Figure 11). Given that *tgs1*Δ was lethal with *YHC1-(1-170)*, we invoke genetic redundancy of Yhc1-(171-189) with the TMG cap.

On the severe end of the synergy spectrum, *YHC1-(1-189)* was lethal when combined with U1 snRNA allele *U1-[+1]*. Thus, the Yhc1 segment from aa 190 to 212 is uniquely essential in this genetic background, among those tested. When combined with other U1 snRNA variants, *YHC1-(1-189)* displayed a ts growth defect with *U1-H1L* (Figure 10, bottom panel) and a cs slow growth defect with *U1+25*, although thriving with *U1-U^5^C*. Collectively, our findings indicate that the Yhc1 C-terminal domain buffers the impact of diverse hypomorphic mutations at the 5′ end of the U1 snRNA.

Finally, we tested the three active Yhc1 C-terminal truncation mutants for their ability to bypass *prp28*Δ and found that none was able to do so (not shown). Thus, the *prp28*Δ bypass effect appears to be specific to mutations in the globular N-terminal domain of Yhc1.

## DISCUSSION

The present study comprises a mutational analysis of the essential U1 snRNP subunit Yhc1, guided in large part by the NMR structure of the homologous U1C subunit of the human U1 snRNP. Deletion analysis and alanine scanning revealed a surprising (to us) high tolerance for subtraction of Yhc1 structural elements, whereby removal of the C-terminal 61 aa had no impact on yeast growth nor did alanine substitutions for any of the nine conserved and surface-exposed amino acids in the Yhc1 N-terminal domain. Even the signature zinc-binding site in the globular N-terminal domain tolerated non-lethal alanine changes at three of the four zinc ligands.

The low-resolution crystal structure of human U1 snRNP containing the N-terminal domain of the U1C subunit (10) revealed that the globular fold nucleated by the tetrahedral zinc complex (rendered as a light green surface model in Figure 8A) is in proximity to the conserved single-stranded U1 snRNA 5′ leader sequence ^1^AUACUUACCU^10^. In the crystal, the U1 leader is annealed to another RNA strand from an adjacent U1 RNA to form a short duplex that mimics the base-paired segment formed by the conserved U1 snRNA ^3^ACUUAC^8^ motif (depicted as blue spheres for phosphorus atoms in Figure 8A) and the 5′SS of the pre-mRNA intron (rendered as beige spheres for phosphorus atoms in Figure 8A). In Figure 8, we superimposed the NMR structure of the U1C globular fold on the low-resolution envelope for U1C in the U1 snRNP crystal structure (pdb 3CW1, which does not include atomic models of the amino acid side chains or RNA nucleotides). This model implicates several of the conserved side chains mutated presently as potential contact points to the intron 5′SS, the U1 snRNA TMG cap or the H1 helix. Apparently, none of these putative interactions is essential *per se* for U1 snRNP function.

### Novel genetic interactions of the Yhc1 N-domain mutations

Deeper insights emerged from a survey of the collection of fully active Yhc1 mutants for synthetic loss of function phenotypes in nine different genetic contexts: in the absence of the inessential U1 snRNP subunits Nam8 and Mud1; in the absence of the TMG cap on U1 snRNA (and other snRNAs); in the absence of the Mud2 subunit of the Msl5•Mud2 branchpoint binding protein complex and in the presence of five different biologically active U1 snRNAs with mutations in or flanking the U1 segment that base-pairs with the 5′SS of the pre-mRNA intron. The mutational synergies were many and notable for a hierarchy of severity and allele specificity, as summarized in Supplementary Figure S5.

The R21A mutation in Yhc1 elicited the most severe and widespread effects, being lethal in the absence of Mud2, severely sick in the absence of Nam8 or the TMG cap and mildly sick in the absence of Mud1. The R21A lesion also conferred lethality of severe sickness in the presence of all five U1 snRNA variants tested. We inferred from the superimposed U1 snRNP and U1C structures that Yhc1 Arg21 contacts the SmD3 Glu37 and/or Asp38 side chains, and that loss of these contacts might account for the synthetic growth defects of *YHC1-R21A* and its ability to bypass *prp28*Δ. Consistent with this hypothesis, we found that mutating SmD3 Glu37 and Asp38 to alanine had no effect on growth *per se* but mimicked several of the phenotypes seen for *YHC1-R21A*, including *prp28*Δ bypass, near-lethality with *mud2*Δ and failure to grow at 37°C in the *mud1*Δ background. The genetics implicates the Yhc1(R21)•SmD3 interface in stabilizing the U1•pre-mRNA•[Msl5•Mud2] early spliceosome intermediate.

The next most consequential lesion in Yhc1, with respect to synergies, was K28A, which was lethal with *U1-ΔU^10^* and severely sick with *U1-[+1]* and *mud2*Δ (Supplementary Figure S5). The equivalent Arg28 side chain in human U1C is poised to interact with the pre-mRNA intron 5′SS:U1 snRNA duplex (Figure 8A). We attribute the various synthetic phenotypes of *YHC1-K28A*, and its ability to bypass the lethality of *prp28*Δ, to the loss of a Yhc1•RNA interaction that in turn affects the stability of the U1•5′SS complex.

Alanine substitutions for Tyr12 or Thr14 caused strong synthetic growth defects in diverse genetic backgrounds (Supplementary Figure S5). Tyr12 and Thr14 in human U1C are located near the human U1 snRNA (Figure 8A), at the junction of the H1 helix and the duplex stem of the stem-loop 3 (SL3) secondary structure (10). It makes sense that loss of Tyr12 and Thr14 is deleterious in the *U1-UΔ^10^* background, where the distance between the ^3^ACUUAC^8^ motif that pairs with the 5′SS and the start of the H1 helix is shortened. The synergy of *Y12A* and *T14A* with *mud2Δ* suggests that these lesions also impact cross-intron interactions of the U1 snRNP with the branchpoint-binding protein.

The *YHC1-H15A* allele was severely sick in only one of the genetic backgrounds tested, *U1-ΔU^10^*, and was mildly sick with *U1-[+1]* in which an extra nucleotide is inserted between C^8^ and C^9^ (Supplementary Figure S5). These results resonate with the crystal structure of the U1 snRNP and the aligned U1C N-domain, which places His15 near the short U1 snRNA segment that connects the ^3^ACUUAC^8^ motif to the start of the H1 helix. The structures suggest that His15 contacts nucleotide 11 of the U1 snRNA (G^11^ in human U1; U^11^ in yeast U1).

### Synergies of Yhc1 C-domain mutations

The C-terminal half of U1C is not present in the crystal structure of the human U1 snRNP. Lack of conservation of this segment in U1C and yeast Yhc1 hints that they might have distinct functions dictated by the significant differences in the RNA and protein composition of the human and yeast U1 snRNPs. By conducting an analysis of the effects of C-terminal truncations of yeast Yhc1, we traced a wide network of genetic interactions between inessential segments of the Yhc1 C-terminal domain and all other components of the early spliceosome that were interrogated here, with the notable exception of Nam8. The *nam8*Δ allele is itself synthetic lethal or severely sick in the absence of Mud1, Mud2 or TMG caps, or when combined with benign mutations in U1 snRNA or an essential splicing factor (17*,*^21^*,*^25^*,*^26^*,*^32–34^*,*^49^), including, as shown here, with the N-terminal R21A mutation in Yhc1. The fact that *nam8*Δ has no effect when combined with the *YHC1-(1-170)*, and that *nam8*Δ and *YHC1-(1-170)* have a similar spectrum of synergies with other spliceosome components, suggest that they are epistatic, i.e. Nam8 and the Yhc1 C-terminal domain act in the same (genetically buffered) pathway during spliceosome assembly.

## SUPPLEMENTARY DATA

Supplementary Data are available at NAR Online.

## FUNDING

National Institutes of Health (NIH) [GM52470 to S.S. and GM102961 to B.S.]. Funding for open access charge: NIH [GM52470].

*Conflict of interest statement*. None declared.

## Supplementary Material

Supplementary Data

## References

[1] Fabrizio P, Dannenberg J, Dube P, Kastner B, Stark H, Urlaub H, Lührmann R (2009). The evolutionarily conserved core design of the catalytic activation step of the yeast spliceosome. Mol. Cell.

[2] Warlocki Z, Odenwälder P, Schitzova J, Platzmann F, Stark H, Urlaub H, Ficner R, Fabrizio P, Lührmann R (2009). Reconstitution of both steps of *Saccharomyces cerevisiae* splicing with purified spliceosomal components. Nat. Struct. Mol. Biol..

[3] Hoskins AA, Friedman LJ, Gallagher SS, Crawford DJ, Anderson EG, Wombacher R, Ramirez N, Cornish VW, Gelles J, Moore MJ (2011). Ordered and dynamic assembly of single spliceosomes. Science.

[4] Chen JY, Stands L, Staley JP, Jackups RR, Latus LJ, Chang TH (2001). Specific alterations of U1-C protein or U1 small nuclear RNA can eliminate the requirement of Prp28p, an essential DEAD box splicing factor. Mol. Cell.

[5] Staley JP, Guthrie C (1999). An RNA switch at the 5′ splice site requires ATP and the DEAD box protein Prp28p. Mol. Cell.

[6] Tang J, Abovich N, Fleming MJ, Séraphin B, Rosbash M (1997). Identification and characterization of a yeast homolog of U1 snRNP-specific protein C. EMBO J..

[7] Gottschalk A, Tang J, Puig O, Salgado J, Neubauer G, Colot HV, Mann M, Séraphin B, Rosbash M, Lührmann R (1998). A comprehensive biochemical and genetic analysis of the yeast U1 snRNP reveals five novel proteins. RNA.

[8] Fortes P, Bilbao-Cortés D, Fornerod M, Rigaut G, Raymond W, Séraphin B, Mattaj IW (1999). Luc7p, a novel yeast U1 snRNP protein with a role in 5′ splice site recognition. Genes Dev..

[9] Schwer B, Erdjument-Bromage H, Shuman S (2011). Composition of yeast snRNPs and snoRNPs in the absence of trimethylguanosine caps reveals nuclear cap binding protein as a gained U1 component implicated in the cold-sensitivity of tgs1Δ cells. Nucleic Acids Res..

[10] Pomeranz Krummel DA, Oubridge C, Leung AKW, Li J, Nagai K (2009). Crystal structure of human spliceosomal U1 snRNP at 5.5 Å resolution. Nature.

[11] Weber G, Trowitzsch S, Kastner B, Lührmann R, Wahl MC (2010). Functional organization of the Sm core in the crystal structure of human U1 snRNP. EMBO J..

[12] Van der Feltz C, Anthony K, Brilot A, Pomeranz Krummel DA (2012). Architecture of the spliceosome. Biochemistry.

[13] Siliciano PG, Guthrie C (1988). 5′ splice site selection in yeast: genetic alterations in base-pairing with U1 reveal additional requirements. Genes Dev..

[14] Séraphin B, Kretzner L, Rosbash M (1988). A U1 snRNA:pre-mRNA base pairing interaction is required early in yeast spliceosome assembly but does not uniquely define the 5′ cleavage site. EMBO J..

[15] Siliciano PG, Kivens WJ, Guthrie C (1991). More than half of yeast U1 snRNA is dispensable for growth. Nucleic Acids Res..

[16] Liao X, Kretzner L, Séraphin B, Rosbash M (1990). Universally conserved and yeast-specific U1 snRNA sequences are important but not essential for U1 snRNP function. Genes Dev..

[17] Hausmann S, Zheng S, Costanzo M, Brost RL, Garcin D, Boone C, Shuman S, Schwer B (2008). Genetic and biochemical analysis of yeast and human cap trimethylguanosine synthase: functional overlap of TMG caps, snRNP components, pre-mRNA splicing factors, and RNA decay pathways. J. Biol. Chem..

[18] Liao XC, Tang J, Rosbash M (1991). An enhancer screen identifies a gene that encodes the yeast U1 snRNP A protein: implications for snRNP protein function in pre-mRNA splicing. Genes Dev..

[19] Abovich N, Liao XC, Rosbash M (1994). The yeast MUD2 protein: an interaction with PRP11 defines a bridge between commitment complexes and U2 snRNP addition. Genes Dev..

[20] Chang J, Schwer B, Shuman S (2010). Mutational analyses of trimethylguanosine synthase (Tgs1) and Mud2: proteins implicated in pre-mRNA splicing. RNA.

[21] Qiu ZR, Schwer B, Shuman S (2011). Determinants of Nam8-dependent splicing of meiotic pre-mRNAs. Nucleic Acids Res..

[22] Hilleren PJ, Kao HY, Siliciano PG (1995). The amino-terminal domain of yeast U1-70K is necessary and sufficient for function. Mol. Cell. Biol..

[23] Ester C, Uetz P (2008). The FF domains of yeast U1 snRNP protein Prp40 mediate interactions with Luc7 and Snu71. BMC Biochem..

[24] Görnemann J, Barrandon C, Hujer K, Rutz B, Rigaut G, Kotovic KM, Faux C, Neugebauer KM, Séraphin B (2011). Cotranscriptional spliceosome assembly and splicing are independent of the Prp40p WW domain. RNA.

[25] Schwer B, Chang J, Shuman S (2013). Structure-function analysis of the 5′ end of yeast U1 snRNA highlights genetic interactions with the Msl5•Mud2 branchpoint binding complex and other spliceosome assembly factors. Nucleic Acids Res..

[26] Chang J, Schwer B, Shuman S (2012). Structure-function analysis and genetic interactions of the yeast branchpoint binding protein Msl5. Nucleic Acids Res..

[27] Colot HV, Stutz F, Rosbash M (1996). The yeast splicing factor Mud13p is a commitment complex component and corresponds to CBP20, the small subunit of the nuclear cap-binding complex. Genes Dev..

[28] Abovich N, Rosbash M (1997). Cross-intron bridging interactions in the yeast commitment complex are conserved in mammals. Cell.

[29] Tang J, Abovich N, Rosbash M (1996). Identification and characterization of a yeast gene encoding the U2 small nuclear ribonucleoprotein particle B” protein. Mol. Cell. Biol..

[30] Fortes P, Kufel J, Fornerod M, Polycarpou-Schwarz M, Lafontaine D, Tollervey D, Mattaj IW (1999). Genetic and physical interaction involving the yeast nuclear cap-binding complex. Mol. Cell. Biol..

[31] Tong AH, Evangelista M, Parsons AB, Xu H, Bader GD, Page N, Robinson M, Raghibizadeh S, Hogue CW, Bussey H (2001). Systematic genetic analysis with ordered arrays of yeast deletion mutants. Science.

[32] Wilmes GM, Bergkessel M, Bandyopadhyay S, Shales M, Braberg H, Cagney G, Collins SR, Whitworth GB, Kress TL, Weissman JS (2008). A genetic interaction map of RNA-processing factors reveals links between Sem1/Dss1-containing complexes and mRNA export and splicing. Mol. Cell.

[33] Costanzo M, Baryshnikova A, Bellay J, Kim Y, Spear ED, Sevier CS, Ding H, Koh JL, Toufighi K, Mostafavi S (2010). The genetic landscape of a cell. Science.

[34] Qiu ZR, Chico L, Chang J, Shuman S, Schwer B (2012). Genetic interactions of hypomorphic mutations in the m7G cap binding pocket of yeast nuclear cap binding complex: an essential role for Cbc2 in meiosis via splicing of MER3 pre-mRNA. RNA.

[35] Calero G, Wilson KF, Ly T, Rios-Steiner JL, Clardy JC, Cerione RA (2002). Structural basis of m7GpppG binding to the nuclear cap-binding protein complex. Nat. Struct. Biol..

[36] Mazza C, Segref A, Mattaj IW, Cusack S (2002). Large-scale induced fit recognition of an m7GpppG cap analogue by the human nuclear cap-binding complex. EMBO J..

[37] Liu Z, Luyten I, Bottomley MJ, Messias AC, Houngninou-Molango S, Sprangers R, Zanier K, Krämer A, Sattler M (2001). Structural basis for recognition of the intron branch site RNA by splicing factor 1. Science.

[38] Muto Y, Pomeranz Krummel D, Oubridge C, Hernandez H, Robinson CV, Neihaus D, Nagai K (2004). The structure and biochemical properties of the human spliceosomal protein U1C. J. Mol. Biol..

[39] Nelissen RLH, Heinrichs V, Habets WJ, Simons F, Lührmann R, van Venrooij WJ (1991). Zinc finger-like structure in U1-specific protein C is essential for specific binding to U1 snRNP. Nucleic Acids Res..

[40] Zhang D, Rosbash M (1999). Identification of eight proteins that cross-link to pre-mRNA in the yeast commitment complex. Genes Dev..

[41] Du H, Rosbash M (2002). The U1 snRNP protein U1C recognizes the 5′ splice site in the absence of base pairing. Nature.

[42] Will CL, Rümpler S, Gunnewiek JK, van Venrooij WJ, Lührmann R (1996). *In vitro* reconstitution of mammalian U1 snRNPs active in splicing: the U1-C protein enhances the formation of early (E) spliceosomal complexes. Nucleic Acids Res..

[43] Mouaikel J, Verheggen C, Bertrand E, Tazi J, Bordonné R (2002). Hypermethylation of the cap structure of both yeast snRNAs and snoRNAs requires a conserved methyltransferase that is localized to the nucleolus. Mol. Cell.

[44] Hausmann S, Shuman S (2005). Specificity and mechanism of RNA cap guanine-N2 methyltransferase (Tgs1). J. Biol. Chem..

[45] Strauss EJ, Guthrie C (1994). PRP28, a DEAD-box protein, is required for the first step of mRNA splicing *in vitro*. Nucleic Acids Res..

[46] Chang TH, Latus LJ, Liu Z, Abbott JM (1997). Genetic interactions of conserved regions in the DEAD-box protein Prp28p. Nucleic Acids Res..

[47] Hage R, Tung L, Du H, Stands L, Rosbash M, Chang TH (2009). A targeted bypass screen identifies Ynl187p, Prp42p, Snu71p, and Cbp80p for stable U1 snRNP/pre-mRNA interaction. Mol. Cell. Biol..

[48] Sengoku T, Nureki O, Nakamura A, Kobayashi S, Yokoyama S (2006). Structural basis for RNA unwinding by the DEAD-box protein *Drosophila* Vasa. Cell.

[49] Balzer RJ, Henry MF (2008). Snu56p is required for Mer1p-activates meiotic splicing. Mol. Cell. Biol..

