# Supplementary material for: Structure-function analysis of the Yhc1 subunit of yeast U1 snRNP and genetic interactions of Yhc1 with Mud2, Nam8, Mud1, Tgs1, U1 snRNA, SmD3 and Prp28
Source: Nucleic Acids Res. 2014 Jan 31;42(7):4697–711. doi: 10.1093/nar/gku097 (PMC3985668; doi:10.1093/nar/gku097)
Supplement: Supplementary Data [file supp_42_7_4697__index.html]

Structure-function analysis of the Yhc1 subunit of yeast U1 snRNP and genetic interactions of Yhc1 with Mud2, Nam8, Mud1, Tgs1, U1 snRNA, SmD3 and Prp28 — Structure-function analysis of the Yhc1 subunit of yeast U1 snRNP and genetic interactions of Yhc1 with Mud2, Nam8, Mud1, Tgs1, U1 snRNA, SmD3 and Prp28 — Supplementary Data 

# Structure-function analysis of the Yhc1 subunit of yeast U1 snRNP and genetic interactions of Yhc1 with Mud2, Nam8, Mud1, Tgs1, U1 snRNA, SmD3 and Prp28

## Supplementary Data

files

**Files in this Data Supplement:**

- Supplementary Data - pdf file
