# Supplementary material for: Structure-function analysis of the Yhc1 subunit of yeast U1 snRNP and genetic interactions of Yhc1 with Mud2, Nam8, Mud1, Tgs1, U1 snRNA, SmD3 and Prp28
Source: Nucleic Acids Res. 2014 Jan 31;42(7):4697–711. doi: 10.1093/nar/gku097 (PMC3985668; doi:10.1093/nar/gku097)
Supplement: Supplementary Data [file supp_gku097_nar-02906-a-2013-File013.pdf]

Supplementary Material

Structure-function analysis of the Yhc1 subunit of yeast U1 snRNP and genetic interactions of Yhc1 with Mud2, Nam8, Mud1, Tgs1, U1 snRNA, SmD3 and Prp28

Beate Schwer and Stewart Shuman

Figures S1, S2, S3, S4, S5

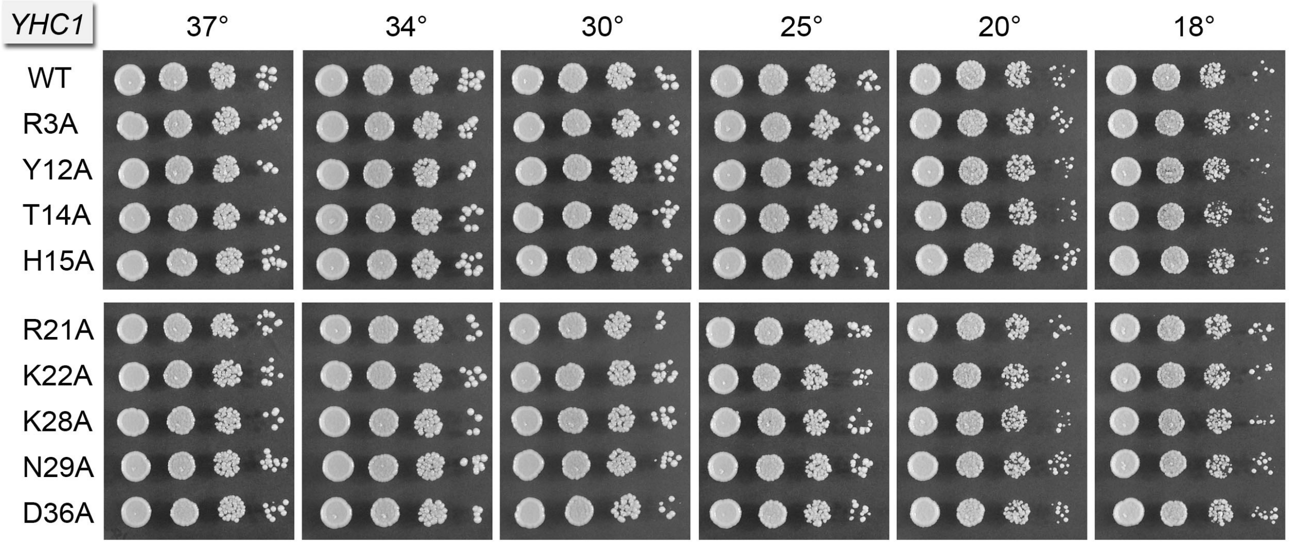

Figure S1. **Effects of alanine mutations on Yhc1 function *in vivo***. The *yhc1*Δ p[*CEN HIS3 YHC1*] strains obtained after plasmid shuffle, bearing the *YHC1* alleles indicated at left, were spot tested for growth on YPD agar at the temperatures specified.

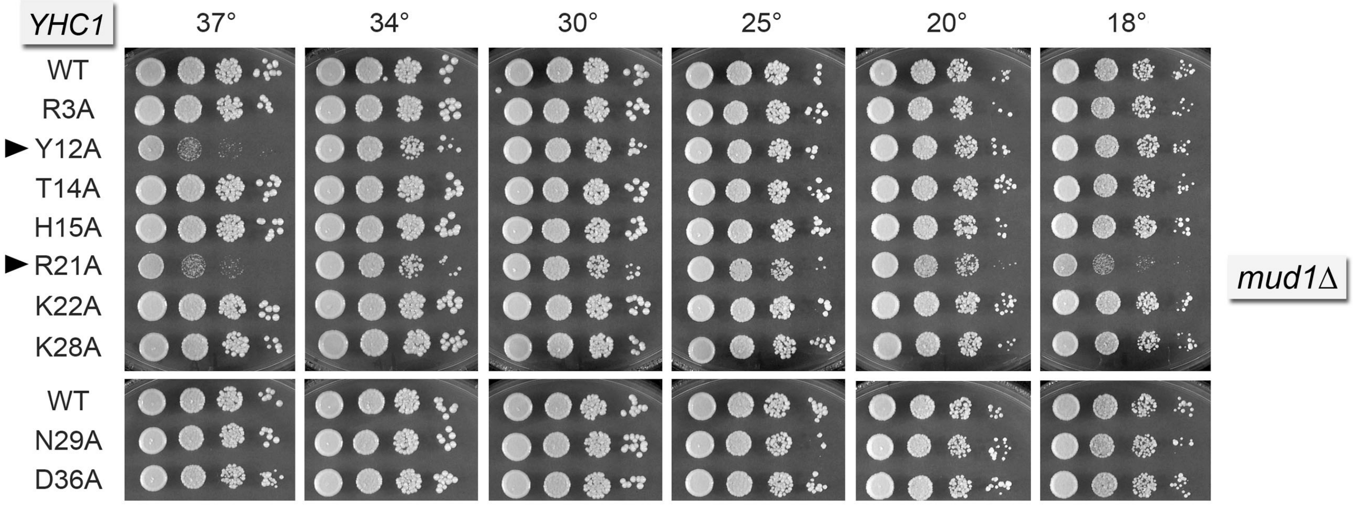

Figure S2. **Synthetic interactions of Yhc1-Ala mutants with Mud1**. Yeast *mud1*Δ *yhc1*Δ strains bearing the indicated *YHC1* alleles were spot-tested for growth at the temperatures specified. The *Y12A* and R21A alleles that displayed synthetic sickness at one or both extremes of temperature are indicated by ▶ at *left*.

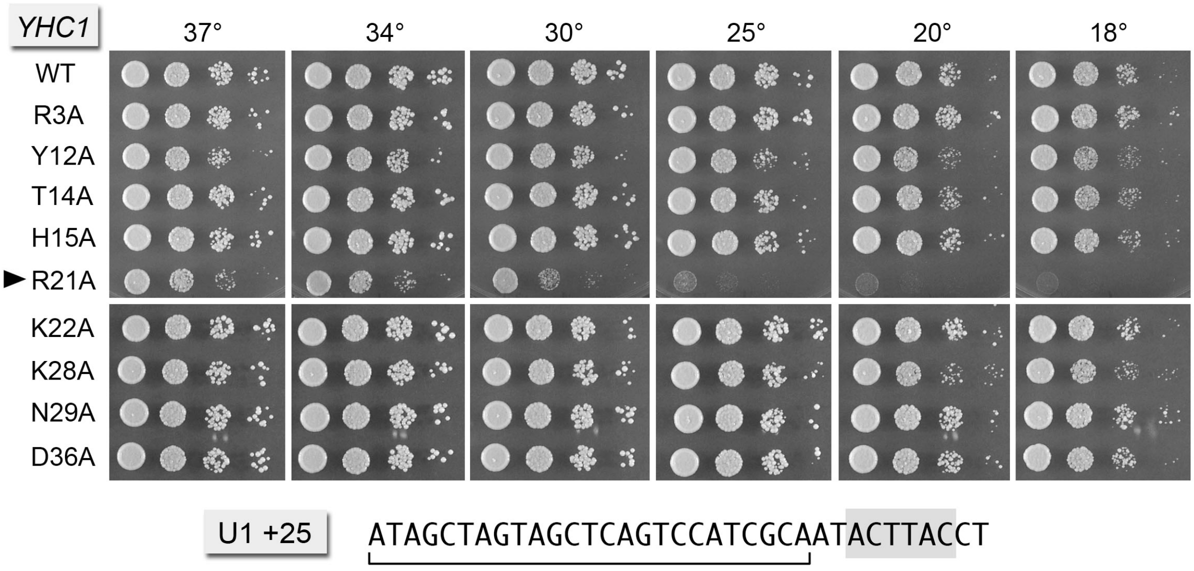

Figure S3. **Synthetic interaction of *YHC1-R21A* with U1 snRNA 5'-extension *U1+25***. The DNA sequence encoding the 5' end of the U1+25 sRNA is shown at *bottom*. The extra 25-nucleotide spacer segment between the 5'-TMG cap and native U1 sequence is demarcated by the bracket. The segment encoding the ^{3}ACUUAC^{8} motif that pairs with the intron 5'SS is highlighted in grey. The *U1+25 yhc1*Δ strains bearing the indicated *YHC1* alleles were spot-tested for growth at the temperatures specified. The synthetic growth defect of the *R21A* allele is denoted by ▶ at *left*.

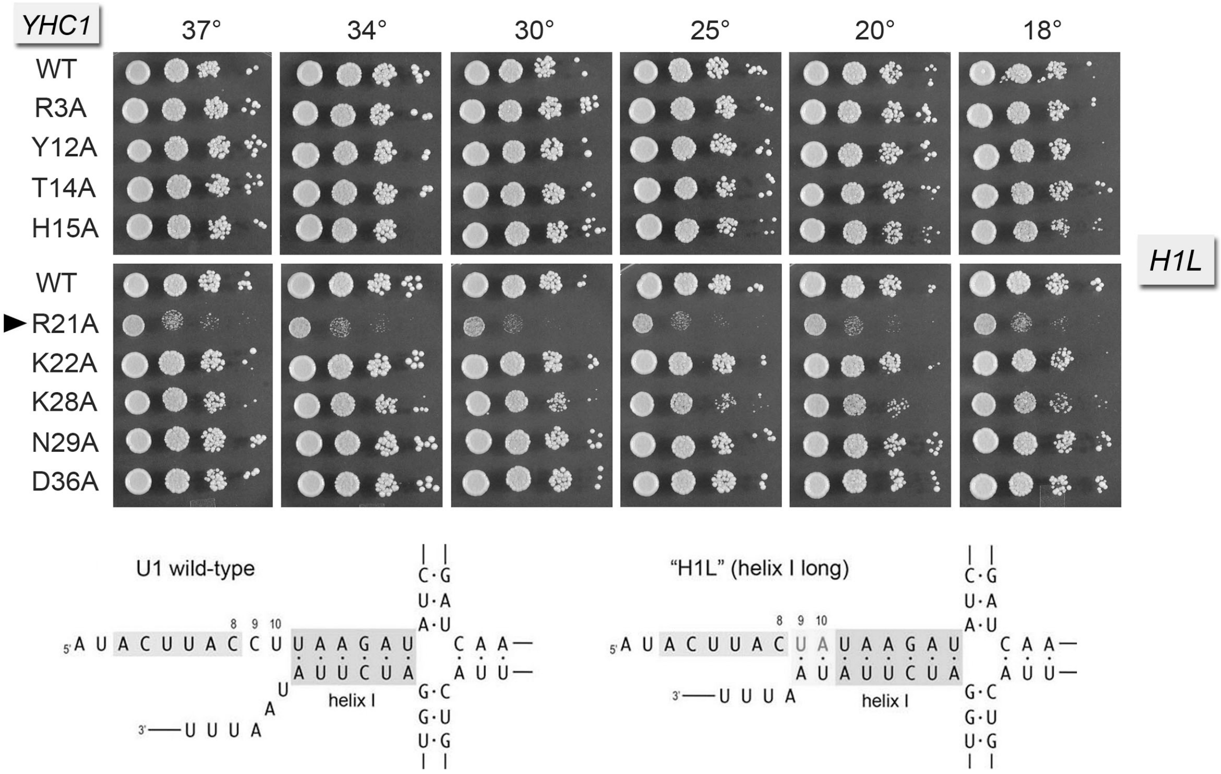

Figure S4. **Synthetic interaction of Yhc1-R21A with U1 snRNA "helix 1 long" mutant**. The primary and predicted secondary structures at the 5' end of wild-type U1 RNA and the H1L ("helix 1 long") mutant are shown at *bottom*. The ^{3}ACUUAC^{8} segment that pairs with the intron 5'SS is highlighted in light grey. Helix 1 is shaded dark grey. The mutation of ^{9}CU^{10} to ^{9}UA^{10} extends helix 1 by two base pairs. The *U1-H1L yhc1*Δ strains bearing the indicated *YHC1* alleles were spot-tested for growth at the temperatures specified. The strong synthetic growth defect of the *R21A* allele is denoted by ▶ at *left*.

	R21A	*K28A*	*Y12A*	*T14A*	*K22A*	*H15A*	*N29A*	*D36A*
mud2Δ	lethal	very sick	very sick	sick				
nam8Δ	very sick							
tgs1Δ	very sick	sick	very sick	sick				
mud1Δ	sick		sick					
prp28Δ	bypass	bypass						
U1-U^{5}C	very sick		sick					
U1+25	very sick							
U1-H1L	very sick							
U1-[+1]	very sick	very sick		sick	very sick	sick		
U1-ΔU^{10}	lethal	lethal	very sick	lethal	very sick	very sick	very sick	↑↑

Figure S5. **Summary of mutational synergies of Yhc1-Ala mutants**. Synthetically lethal pairs of alleles are highlighted in red boxes. Other negative pairwise interactions are classified as very sick (yellow boxes) or mildly sick (light green boxes). Allelic pairs that resulted in gain-of-function genetic effects are highlighted in blue boxes, including bypass of the lethality of *prp28Δ* by *R21A* and *K28A* and suppression of the growth defect of *U1-ΔU^{10}* by *D36A*.
